# Systems identification and characterization of β-glucuronosyltransferase genes involved in arabinogalactan-protein biosynthesis in plant genomes

**DOI:** 10.1038/s41598-020-72658-4

**Published:** 2020-11-25

**Authors:** Oyeyemi Olugbenga Ajayi, Allan M. Showalter

**Affiliations:** 1 Department of Environmental and Plant Biology, Ohio University, Athens, 45701 USA; 2 Molecular and Cellular Biology Program, Ohio University, Athens, 45701 USA

**Keywords:** Biochemistry, Computational biology and bioinformatics, Evolution, Genetics, Molecular biology, Plant sciences, Systems biology

## Abstract

Utilizing plant biomass for bioethanol production requires an understanding of the molecular mechanisms involved in plant cell wall assembly. Arabinogalactan-proteins (AGPs) are glycoproteins that interact with other cell wall polymers to influence plant growth and developmental processes. Glucuronic acid, which is transferred to the AGP glycan by β-glucuronosyltransferases (GLCATs), is the only acidic sugar in AGPs with the ability to bind calcium. We carried out a comprehensive genome-wide analysis of a putative *GLCAT* gene family involved in AGP biosynthesis by examining its sequence diversity, genetic architecture, phylogenetic and motif characteristics, selection pressure and gene expression in plants. We report the identification of 161 putative *GLCAT* genes distributed across 14 plant genomes and a widely conserved GLCAT catalytic domain. We discovered a phylogenetic clade shared between bryophytes and higher land plants of monocot grass and dicot lineages and identified positively selected sites that do not result in functional divergence of GLCATs. RNA-seq and microarray data analyses of the putative *GLCAT* genes revealed gene expression signatures that likely influence the assembly of plant cell wall polymers which is critical to the overall growth and development of edible and bioenergy crops.

## Introduction

Plants have continually evolved adaptive features after moving to land about 400 million years ago in order to be physiologically and functionally suited for their terrestrial habitats^1,2^. The evolution of gene families can facilitate gene family expansion or contraction and is an important mechanism that drives natural variation for adaptation or speciation in green plants^3^. Important evolutionary mechanisms such as gene duplication and whole genome duplication events (polyploidy) and segmental gene deletion has altered gene family sizes across lineages and advanced phenotypic diversity across the plant kingdom^4^. Following gene duplication and loss, adaptation and speciation appear to proceed through a combination of both structural and *cis*-regulatory changes in one or more paralogous genes^5^. Recent advances in sequencing technology have enabled researchers to make significant progress in understanding gene evolution. This provides insights into the underlying connections between the expansion of gene families and evolution of new gene functions^6^.

Arabinogalactan-proteins (AGPs) are plant cell wall glycoproteins with structurally complex, large-branched polysaccharides attached to hydroxyproline (Hyp) residues. They are ubiquitous in bryophytes and higher plants^7,8^ and are implicated in virtually all aspects of plant growth and development^9–11^. AGPs contain approximately 10% protein and 90% sugar, which forms the interactive molecular surface of AGPs which are essential to their biological functions^12–14^. Type II arabinogalactan (AG) polysaccharides of AGPs possesses structural characteristics mainly a β-(1 → 3)-galactan backbone with β-(1 → 6)-linked galactan side chains which are further decorated with galactopyranosyl (Gal*p*) and arabinofuranosyl (Ara*f*) residues as well as with other less abundant residues including rhamnosyl (Rha), fucosyl (Fuc), and glucuronosyl (GlcA; with or without 4-*O*-methylation) residues^15,16^. The formation and assembly of AG sugar moieties are controlled by a glycosylation process catalyzed by glycosyltransferases (GTs). GTs regulate the sequence and length of the AG chains and act in a regio- and stereo-specific manner^17^. It is speculated that at least ten functionally distinct GTs are required for the biosynthesis of type II AG^11^ and many of these GTs await functional discovery and characterization.

β-glucuronosyltransferases (GLCATs) are involved in the transfer of glucuronic acid (GlcA) to AGPs. Out of the eleven putative β-glucuronosyltransferases (GLCATs) previously discovered in *Arabidopsis thaliana* and assigned to GT14 in the Carbohydrate Active Enzyme (CAZy; https://www.cazy.org/) database, only three GLCATs, namely, AT5G39990 (ATGLCAT14A), AT5G15050 (ATGLCAT14B) and AT2G37585 (ATGLCAT14C) have demonstrated GLCAT catalytic activity based on an in-vitro assay^11^ and contain the conserved Branch domain PF02485 (referred to as GLCAT domain henceforth) present in GT14 gene family. The number and genetic features of putative GLCATs in other plant species remain to be verified despite their fully sequenced genomes. Previous work using nuclear magnetic resonance and molecular dynamics simulations demonstrated that the terminal carboxyl groups of GlcA residues can act as potential intramolecular Ca^2+^-binding sites^18^. Apart from the fact that GlcA residues are the only acidic sugars in the AG chain, their importance in the binding and release of extracellular calcium have been demonstrated^18^. Intuitively, GLCATs may play an essential role in global Ca^2+^ signaling processes in plants and could be a potential target for improving AGP-based products and increasing biomass for biofuel production from bioenergy crops.

The advent of high-throughput technologies in the post-genomic era has generated enormous amounts of data that can yield important biological information for researchers through data mining of publicly available databases^19^. A bioinformatics approach can be an effective tool in understanding gene family expansion, identifying paralogs and orthologs using sequence features, identifying sites under selection pressure and elucidating gene expression dynamics among gene family members. Examination of such data can yield valuable insights that can facilitate and guide further research in the field. Such is the case here, where the availability of whole genome sequences along with microarray, proteome and transcriptome data can enable large-scale investigations into identifying and characterizing gene family members in multiple plant genomes using bioinformatics approaches.

The present study is focused on mining plant genomes for evolutionary footprints of functional importance among putative GLCATs. We carried out a comprehensive analysis aimed at identifying and characterizing the family of enzymes (GLCAT) that transfer GlcA to AG glycan belonging to GT14 family using *Arabidopsis thaliana* GLCAT domain of functionally characterized GLCATs as queries. Our specific goal is to utilize phylogenetic analysis, physical mapping of genes, motif identification, synteny and gene expression analyses to gain insight into the evolution, sequence diversification, functional similarity/divergence, and tissue-specific transcriptional profiling among putative *GLCAT* gene family members in 14 plant genomes.

## Results

### Sequence retrieval, identification and multiple sequence alignments of putative *GLCAT* gene family members in plant lineages

**Figure 1:**
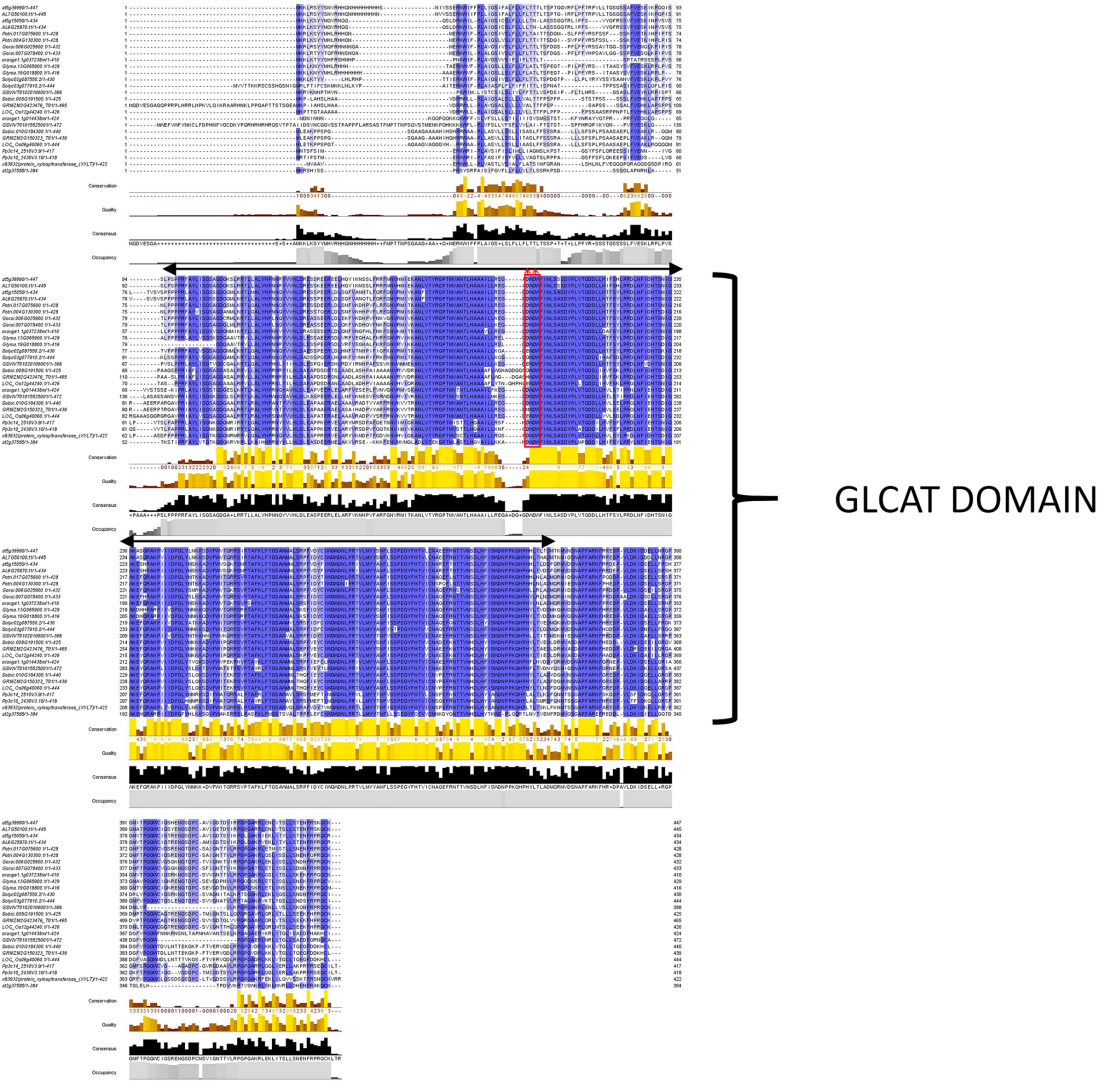
Protein alignment of representative GLCATs from various plant species. Twenty-six proteins representing the diversity of putative GLCATs in the investigated genomes were aligned using ClustalW and illustrated in Jalview^33^. Conserved GLCAT domains are indicated with black arrow lines. The X/DWD/X motif is indicated by the red box region.

**Figure 2:**
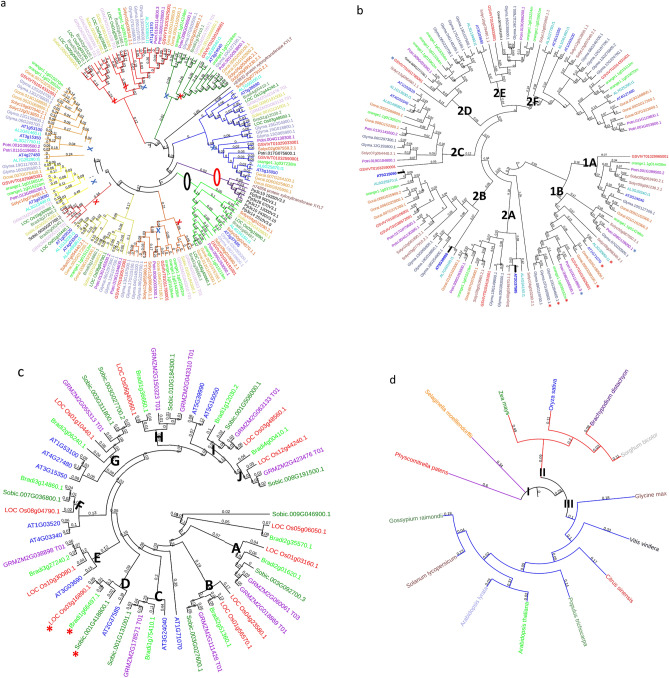
Phylogenetic analyses of putative GLCATs in plant genomes using maximum likelihood method. Figure showed the phylogenetic analyses in 14 plant species investigated (**a**), dicots (**b**), monocot grasses (**c**) and AtGLCAT14A orthologs in 14 plant species (**d**). In (**a**), the phylogenetic tree was created with the full-length GLCAT protein sequences from 14 plant species based on the maximum likelihood method. Ten phylogenetic classes (classes **a**–**j**) were identified. The blue X and red X represent dicot and monocot grass specific clades respectively, corresponding to the different species. The red oval indicates a phylogenetic clade specific to lower land plants while the black oval indicates a clade shared by lower (bryophytes) and higher land plants (monocot grass and dicots). In (**b**), eight phylogenetic clades (classes **1a**, **1b** and **2a**–**f**) were identified. The red and blue asterisks indicated amino acid changes in the second D residue from D → N and D →  T in the DWD consensus motif found in plants respectively. In (**c**), ten phylogenetic clades (clade **a**–**j**) were identified and clades were assigned if it contained minimum of 3 out of the 4 monocot grasses investigated. The red asterisks indicated amino acid changes D → S in the X/DWD/X motif respectively. Arabidopsis GLCAT sequences were included for efficient classification. Genes with no asterisk possess no substitution. In (**d**), the phylogenetic tree was created with the full-length protein sequences of AtGLCAT14A (AT5G39990) and its orthologs in the 14 plant species by the Bayesian Inference using the Bayesian Markov Chain Monte Carlo method as implemented in BEAST software v1.5.4^34^. Support values/posterior probabilities and branch lengths are indicated while I, II and III corresponds to labels for foreground and background in branch and branch-site analyses.

A total of 161 putative *GLCAT* genes were found distributed across 14 plant genomes. Eleven genes were identified as members of the *GLCAT* genes in *Arabidopsis thaliana*, *Arabidopsis lyrata*, *Brachipodium distachyon*, *Oryza sativa* (rice), *Amborella trichopoda* and *Sorghum bicolor* (sorghum). Thirteen *GLCAT* genes were identified in *Citrus sinensis* (orange) and *Populus trichocarpa* (poplar), 22 in *Glycine max* (soybean), 15 in *Solanum lycopersicum* (tomato) and *Gossypium raimondi* (cotton), 6 in *Physcomitrella patens*, 2 in *Selaginella moellendorffii*, 9 in *Vitis vinifera* (grape) and 10 in *Zea mays* (corn) (Supplementary Table S1). Selaginella xylosyltransferase (XYLT) genes appear to be misclassified as it contains sequences that are closer to putative GLCATs than XYLTs. In a blastp analyses, Selaginella sequences had 60.2% sequence identity and 98.8% sequence coverage with ATGLCAT14A compared to 28% sequence identity and 69% coverage with mammalian XYLTs. Also, only the GLCAT domain is present in Selaginella and lacks the XYLT domain present in mammalian GT14 sequences^20^. Each of these genes were confirmed for the presence of the GLCAT domain while excluding sequences having a DUF 266 domain^21^. Multiple alignment of the putative GLCAT protein sequences of representative members of the investigated species identified a widely conserved GLCAT domain which is the catalytic region with less conserved N terminal and C terminal ends following protein sequence alignments (Fig. 1). The DXD motif is a metal ion binding motif present in many glycosyltransferases and this motif is essential to their catalytic functions^20^. There are some representative sequences that appear to have a highly conserved DWD motif with the first D residue less conserved than the second D residue of the motif. In addition, the second D residue of the X/DWD/X motif is substituted with either asparagine (N) or threonine (T) for some dicot species and serine (S) for some monocot grass species (Fig. 2b,c). Surprisingly, we discovered a highly conserved, uncharacterized tryptophan (W) residue in the X/DWD/X motif shared among plant and animal species (Supplementary Fig. S1).

### Phylogenetic analyses of *GLCAT* genes in the investigated plant genomes

The maximum likelihood (ML) method was used to examine the phylogenetic relationships among all 14 plant species (Fig. 2a), including eight dicot species (Fig. 2b) and four monocot grass species (Fig. 2c), which were also examined separately. Similarly, Bayesian Inference (BI) was used to assess the phylogenetic relationships among AtGLCAT14A orthologs and results were compared with those obtained using ML (Fig. 2d). Results obtained from the phylogenetic tree for all species identified a phylogenetic clade (clade E) that is representative of lower and higher land plants (Fig. 2a). Results obtained from the phylogenetic tree for dicot species identified two major clades, namely clades 1 and 2. Clade 1 was subdivided into groups A and B and clade 2 was subdivided into groups A–F (Fig. 2b). Two Arabidopsis thaliana genes, *At3g24040* (group 1A) and *At1g71070* (group 1B) belong to clade 1 while the remaining nine Arabidopsis thaliana genes are present in clade 2. All eight dicot species have orthologs represented in each group (1A-B, 2A-F) except for group 2D which lacks *GLCAT* genes from *Arabidopsis thaliana* and *Arabidopsis lyrata.* No putative GLCAT gene members from a species were clustered into a single group except in *Amborella trichopoda*, whose putative GLCATs occupy distinct positions in the phylogeny analysis (Supplementary Fig. S6). Clades A, B, G, H, I and J contain all species representative of monocot grass species while clades C, D and E clustered with putative *Arabidopsis thaliana GLCAT* genes (Fig. 2c). In class D, the *LOC_Os03g16890.1, Bradi1g66497.1* and *Sobic. 001g418800.1* genes exhibited D to S residue changes in the second D residue of the DWD motif; this residue change is absent in its closest Arabidopsis homolog *AtGLCAT14C* (*At2g37585*) (Fig. 2c). Results of the BI analysis of *AtGLCAT14A* and its orthologs showed a similar clustering pattern that separates monocot grasses from dicots (Fig. 2d).

### Physical mapping, gene structure analysis and synteny analysis

#### Physical mapping of putative *GLCAT* genes in the 14 plant genomes

The physical maps of putative *GLCAT* genes were distributed on all 5 chromosomes in *Arabidopsis thaliana* and seven of the eight chromosomes in *Arabidopsis lyrata.* The largest numbers were observed on chromosome 1 and 3 for *Arabidopsis thaliana* and chromosome 3 for *Arabidopsis lyrata. Solanum lycopersicum* and *Gossypium raimondi* have the same number of *GLCAT* genes distributed across 8 chromosomes in *Solanum lycopersicum* and 9 chromosomes in *Gossypium raimondi.* The largest number of genes were found on chromosomes 12 and 7 for *Solanum lycopersicum* and *Gossypium raimondi* respectively. Similarly, *Vitis vinifera* and *Glycine max GLCAT* genes were distributed over 7 and 12 chromosomes respectively, with the largest number of genes found on chromosomes 1 and 8 for *Vitis vinifera* and chromosomes 6, 12 and 13 for *Glycine max*. Also, putative *GLCAT* genes were distributed over 2 and 6 chromosomes in *Selaginella moellendorffii* and *Physcomitrella patens* respectively (Supplementary Table S1, Supplementary Fig. S2).

#### Gene structure and synteny analyses of putative *GLCAT* genes in the 14 plant genomes

**Figure 3:**
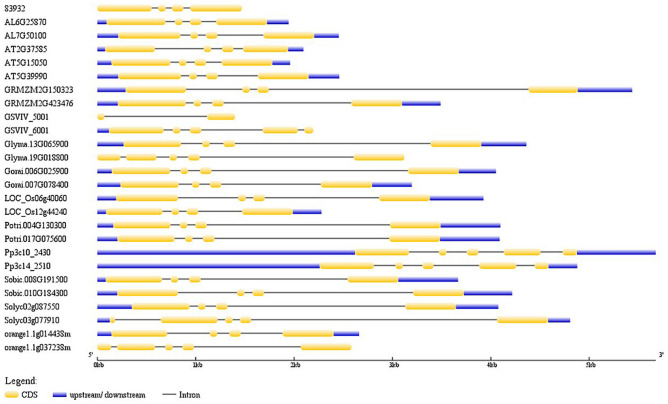
Gene structure display of representative *GLCATs* from various plant species. Twenty-six proteins representing the diversity of putative *GLCATs* in the plant kingdom were used for the gene structure analysis as illustrated by using the GSDS 2.0 server^37^. ‘83,932 = putative *GLCAT* sequence in *Sellaginella moellendorffii*; AL = *Arabidopsis lyrata*; AT = *Arabidopsis thaliana*; GRMZM = *Zea mays*; GSVIV = *Vitis vinifera*; Glyma = *Glycine max*; Gorai = *Gossypium raimondii*; LOC = *Oryza sativa*; Potri = *Populus trichocarpa*; Pp = *Physcomitrella patens*; Sobic = *Sorghum bicolor*; Solyc = *Solanum lycopersicum*; orange = *Citrus sinensis.*

**Figure 4:**
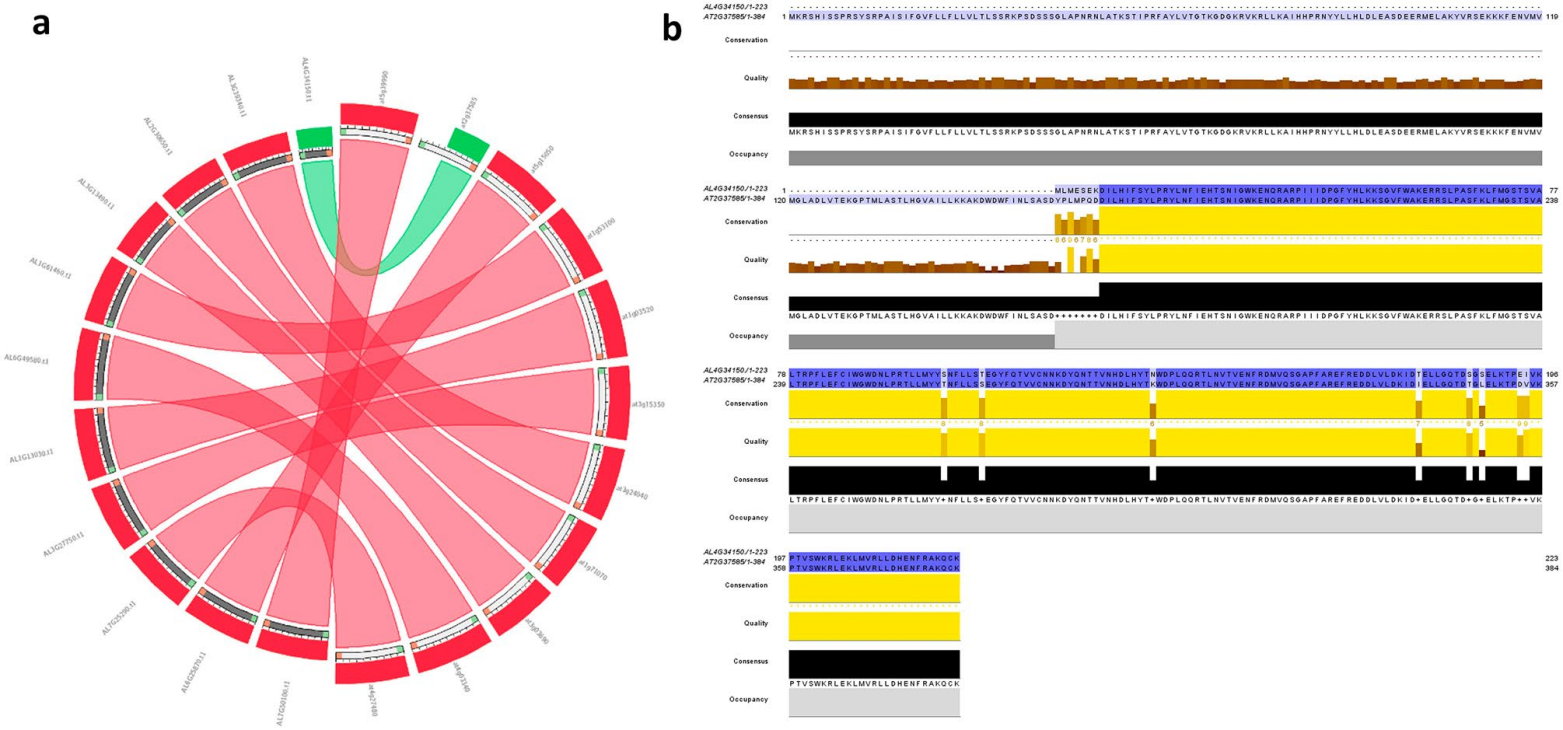
Comparative analyses of *ATGLCAT14C* (*AT2G37585*) and *AL4G34150* genes. (**a**) Synteny of *Arabidopsis thaliana and Arabidopsis lyrata GLCAT* genes. Weak sequence similarity (green ribbon) between *ATGLCAT14C* and its ortholog *AL4G34150* in *A. lyrata.* (**b**) Pairwise comparison between *ATGLCAT14C* and its ortholog *AL4G34150* displaying conserved regions (highlighted in dark blue and yellow blocks) as illustrated by Jalview^33^. The first 167 amino acid residues in *ATGLCAT14C* are conspicuously absent in *AL4G34150.*

Gene structure diversification was studied to gain insights into the evolution of putative *GLCAT* genes in the investigated genomes. Generally, all of the *GLCAT* genes including those of the *Amborella trichopoda* have 4 exons and 3 introns with variable lengths in the 5′ and 3′ UTR regions, except for some representative species of *Glycine max*, *Gossypium raimondi*, *Physcomitrella patens* and *Citrus sinensis* (Fig. 3, Supplementary Fig. S3). Comparative synteny relationship maps displayed a high degree of sequence similarity between *Arabidopsis thaliana GLCAT* gene family members and their respective homologs in other species (Supplementary Fig. S4). Surprisingly, *At2g37585* displayed weak sequence similarity with its closest homolog in *Arabidopsis lyrata* (*Al4g34150*) (Fig. 4).

#### Distribution of amino acid motifs/blocks in putative GLCAT gene family in plant genomes

A total of 161 protein sequences from 14 plant genomes was used to generate a MEME-driven search for conserved amino acid motifs named "blocks". The MEME analysis generated 50 sequence blocks with various lengths ranging from six (blocks 24 and 40) to 41amino acids (blocks 2, 4 and 37) (Supplementary Table S2). Sequence blocks 1–6, 8 and 9 are found in more than 95% of the sequences analyzed (Supplementary Fig. S5). We identified sequence blocks 1, 2, 3, 4, 5, 6, 7, 11 and 12 to be localized to the GLCAT domain. Some sequence blocks are unique to monocot grasses and dicots lineages while some are shared across certain plant lineages (Supplementary Table S2).

#### Positively selected sites in GLCAT14A orthologs and their putative biological significance

The site-specific, branch and branch-site models were used to detect sites under selective pressure among *AtGLCAT14A* orthologs. After removing the gaps, all the amino acid sequences were analyzed using the CodeML program. Results showed that neither the M0 versus M3 or M2a versus M1a models identified positively selected sites. Only the M8 versus M7 model identified several sites with ω values significantly greater than 1 (M8 vs. M7, 2ΔL = 261.53, *p* < 0.001). Eighty-four amino acid sites were identified under positive selection by the M8 alternative model, including 29 amino acid sites that have posterior probability (PP) > 0.95 and 55 sites with PP > 0.99 (Supplementary Table S3). The branch model identified no significant differences between the free ratio model and the one ratio model in all the three branches investigated (Fig. 2d; Supplementary Table S3). According to the likelihood ratio tests (LRT) for the branch site model, comparing model A versus model A null for the branches I, II and III, LRT were significantly different (2ΔlnL = 53.43, *p* = 0.00004 for branch I , 2ΔlnL = 17.92711 , *p* = 0.00002 for branch II; 2ΔlnL = 7.614822, *p* = 0.006 for branch III). In the branch-site model, branch sites had PP < 0.95 based on the Bayes empirical Bayes computation while using each of the branches as foreground and the remaining as backgrounds (Supplementary Table S3).

#### Digital expression analysis of a putative *GLCAT* gene family

**Figure 5:**
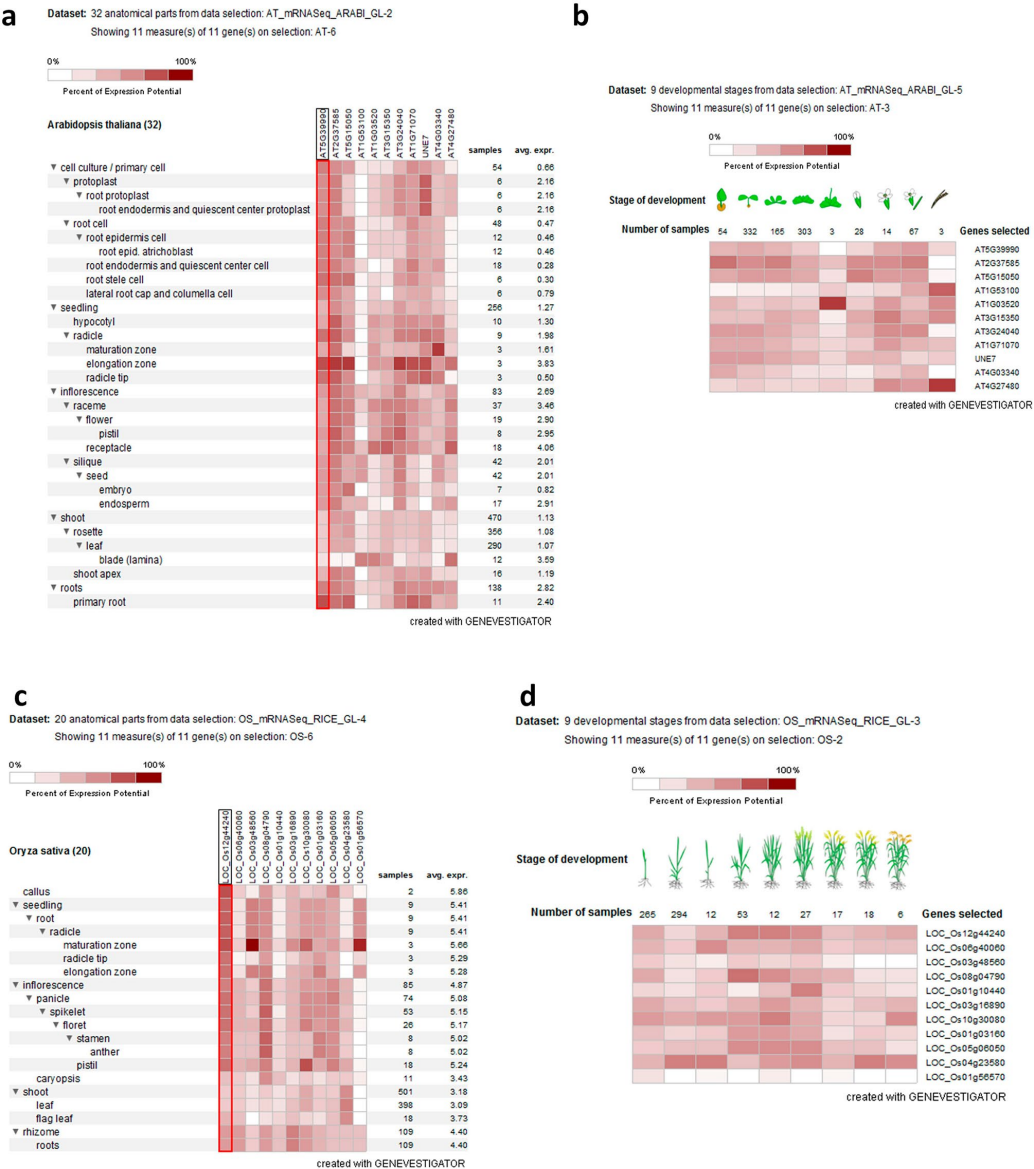

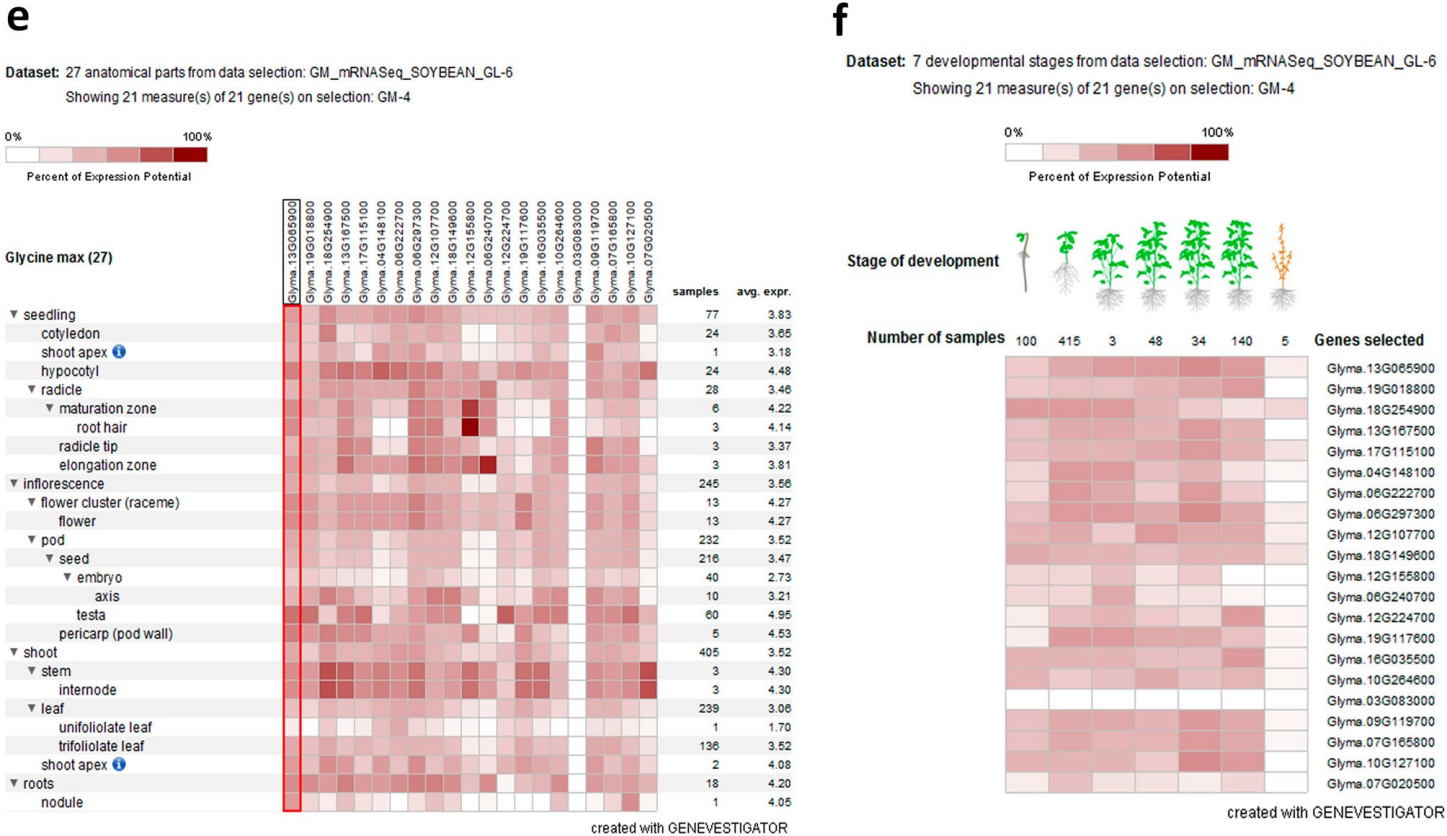
Expression pattern of putative *GLCAT* genes across anatomical reference points and developmental stages in Arabidopsis, rice and soybean. Figure showed the digital expression pattern in the anatomical parts and across developmental stages of Arabidopsis (**a**, **b**), rice (**c**, **d**) and soybean (**e**, **f**). The red boxed region corresponds to the functionally characterized *AtGLCAT14A* (AT5g39990) gene (**a**) and its orthologs in rice (**c**) and soybean (**e**)^11^.

**Figure 6:**
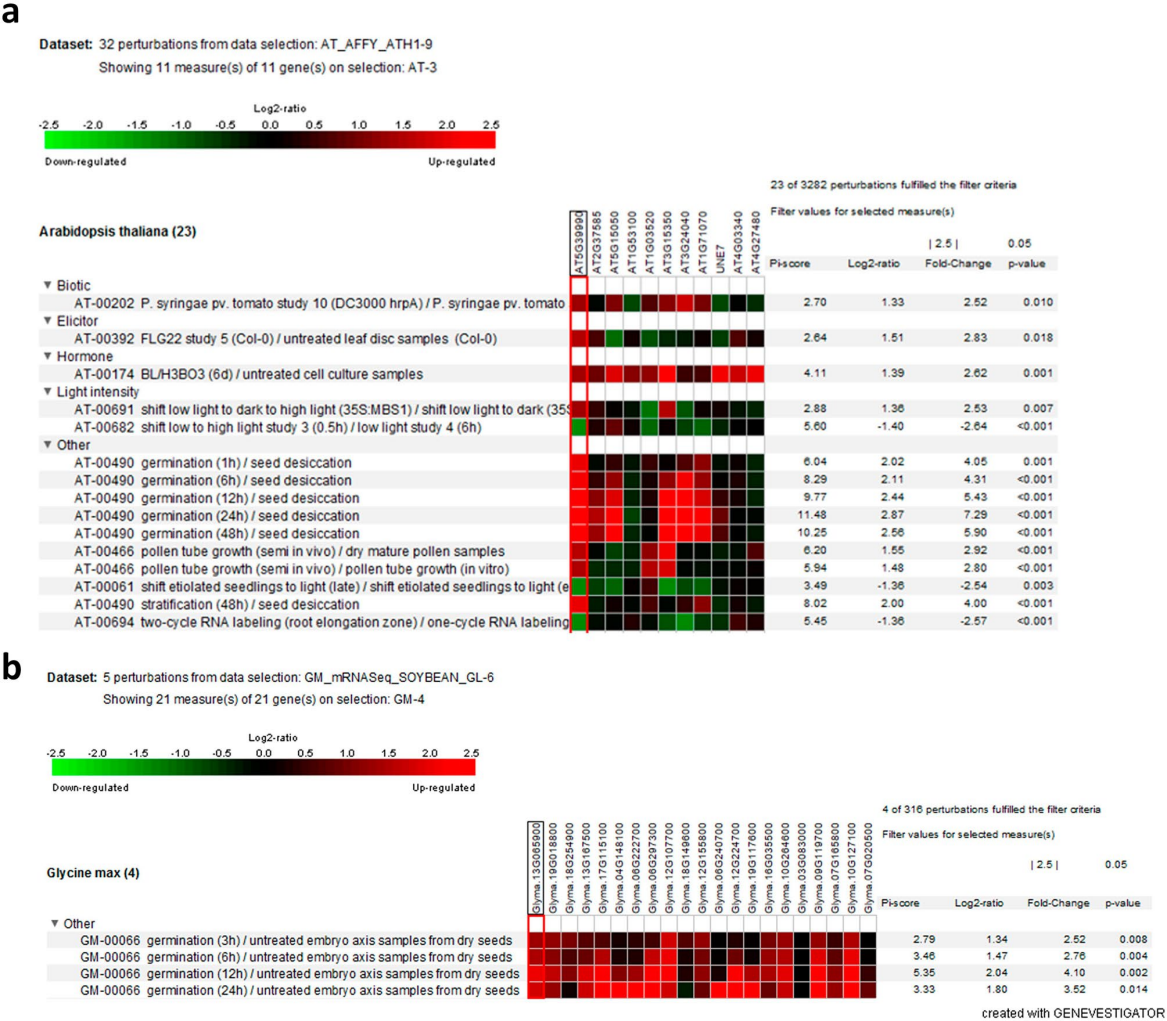
Digital expression pattern of putative *GLCATs* in Arabidopsis and soybean during germination. (**a**) Expression pattern of *GLCAT* genes in *Arabidopsis thaliana* during germination. (**b**) Expression pattern of *GLCAT* genes in soybean during germination. The red boxed regions indicated the A*tGLCAT14A* (AT5g39990) gene and its ortholog in soybean.

The expression pattern among putative *GLCAT* gene family members in *Arabidopsis thaliana* showed differential gene expression across 32 anatomical parts with the highest expression observed in the radical elongation zone (Fig. 5a). Across developmental stages, all Arabidopsis *GLCAT* gene family members had low to moderate expression except in the senescence stage. Interestingly, two genes, *At4g27480* and *At1g53100* have very high expression levels in the senescence stage compared to other stages of development (Fig. 5b). For the expression of *GLCAT* genes during germination in Arabidopsis, microarray data showed that *AtGLCAT14A (At5g39990)* was significantly upregulated and highly expressed (fold change > 2.5) at 1, 6, 12, 24 and 48 h of germination (Fig. 6a). Similarly, significant upregulation (fold change > 2.5) was observed for *AtGLCAT14B (At5g15050)*, *At3g15350*, *At3g24040* and *At1g71070* at 6, 12, 24 and 48hrs of germination (Fig. 6a). Expression profile of *GLCAT* gene family members in *Oryza sativa* identified *LOC_Os12g44240* as expressed in all 20 anatomical parts with differential expression patterns observed among rice *GLCAT* gene members (Fig. 5c). Low to medium expression was observed among the genes; however, two genes, *LOC_Os3g48560* and *LOC_Os1g56570* were highly expressed in the maturation zone of rice root seedlings (Fig. 5c). Differential gene expression was observed across all developmental stages (Fig. 5d). For *GLCAT* gene family members in soybean across 27 anatomical parts, medium expression was observed for most soybean *GLCAT* genes except for *Glyma.03g083000* whose expression was not detected (Fig. 5e). Higher expression during the seedling stage was observed in root hairs for *Glyma.12G155800*, while *Glyma.06G240700* was expressed predominantly in the elongation zone of seedlings (Fig. 5e). Across developmental stages, low to medium expression was observed across *GLCAT* gene family members except for *Glyma.03G083000* which had no detectable expression (Fig. 5f). For soybean *GLCAT* gene family, most of the genes were significantly upregulated (fold change > 2.5) especially at 12 h and 24 h of germination (Fig. 6b). Notably, *Glyma.13g065900*, *Glyma.19g018800*, *Glyma.12g107700*, *Glyma.10g264600*, *Glyma.09g119700*, *Glyma.10g127100* were highly upregulated (fold change > 2.5) at 3 h, 6 h, 12 h and 24 h of germination (Fig. 6b).

#### Digital expression of putative *GLCAT* genes under abiotic stress in *Arabidopsis thaliana* and *Oryza sativa*

**Figure 7:**
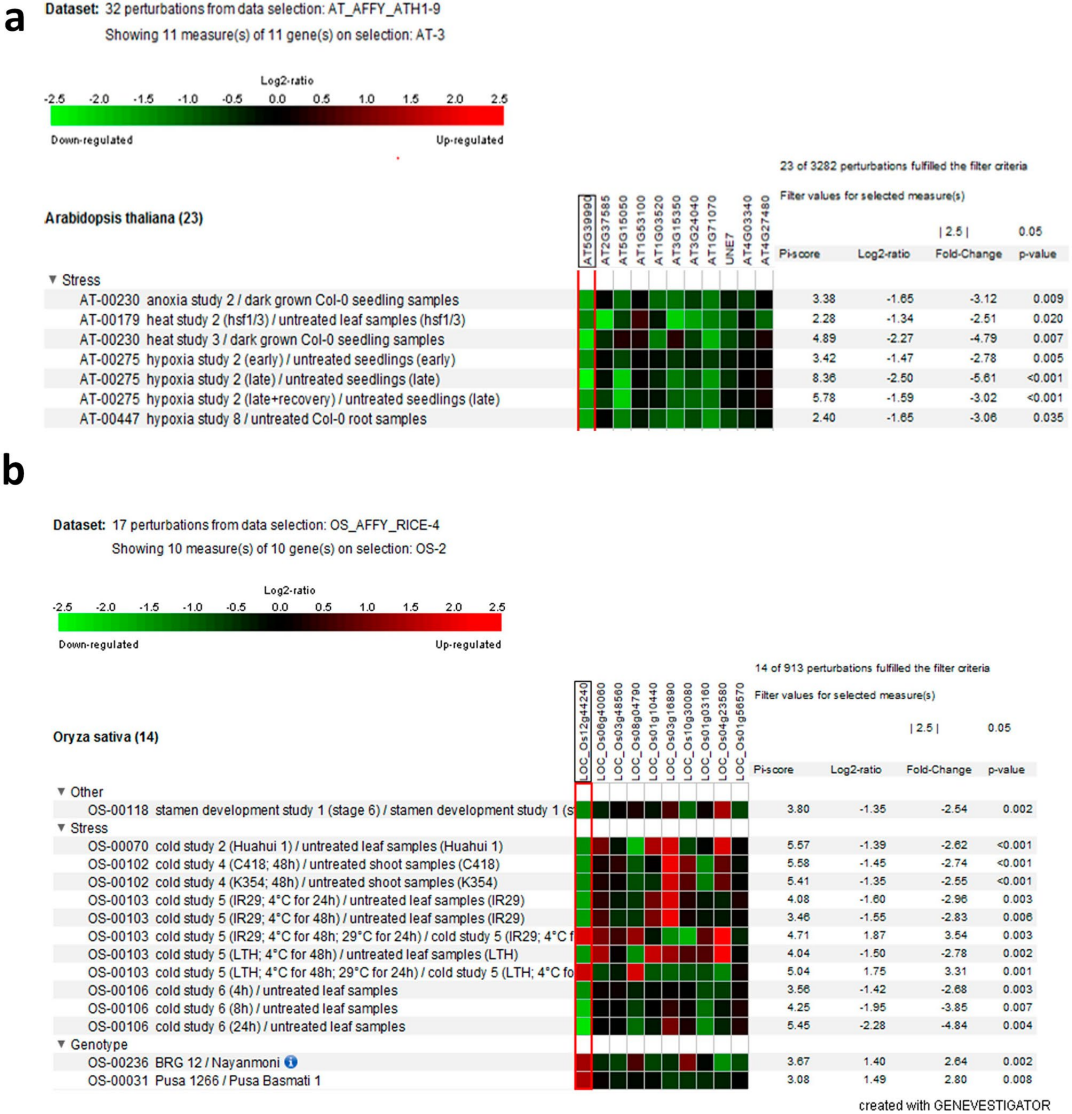
Expression pattern of putative *GLCATs* genes in Arabidopsis and rice in response to abiotic stress. (**a**) Expression pattern of *GLCAT* genes in *Arabidopsis thaliana* during anoxia, heat and hypoxia (**b**) Expression pattern of *GLCAT* genes in rice during cold stress. Note the differential gene expression in all *GLCATs* in Arabidopsis and rice in response to different stressors. The red boxed region indicates AtGlcAT14A (AT5g39990) and its ortholog in rice, both of which are relatively down-regulated in response to stressors.

The modification of plant cell walls is one of the key processes that drives the adaptability of crop species to abiotic stressors. In heat, anoxia and hypoxia studies in *Arabidopsis thaliana*, all the *GLCAT* gene family members either showed no change in expression or were significantly down-regulated (Fig. 7a). Interestingly, *AtGLCAT14A* and *At1g71070* were significantly down-regulated (fold change < 1.5) in all the stressors examined (Fig. 7a). In rice, abiotic expression data was only available for cold stress in specific rice genotypes (Cold tolerance imbred line K354 and its recurrent parent C418, which possesses a cold sensitive phenotype; IR29 and LTH, both chilling-tolerant and chilling-sensitive lines, respectively). For the responses of rice *GLCAT* gene family members to cold stress, *LOC_Os12g44240* was down-regulated (fold change < 2.5) following exposure to cold stress (4 °C) for 24 h and 48 h in shoot and leaf tissues but up-regulated when re-exposed to 29 °C for 24 h only in IR29 (indica) and LTH (japonica) genotypes (Fig. 7b). Surprisingly, *LOC_Os03g16890* was significantly up-regulated (fold change > 1.5) under cold stress but down-regulated during the recovery phase (at 29 °C) in both IR29 and LTH genotypes. Also, *LOC_Os04g23580* was highly up-regulated during the recovery phase (at 29 °C) in IR29 and LTH genotypes while *LOC_Os12g44240* was up-regulated only in IR29 but not in the LTH genotype (Fig. 7b).

## Discussion

AGPs are a complex family of macromolecules with heterogenous sugar chains decorating the protein backbones. AGPs are plant cell wall glycoprotein components with great potential for industrial and food applications, which builds upon their well-known emulsifying, adhesive, and water-holding properties. We considered the plant GT14 gene family as putative GLCATs for the following reasons: (1) The glucuronic acid substitution of xylan (GUX) enzymes have been demonstrated to attach α-GlcA to xylan and the loss of function of *GUX* genes resulted in the complete loss of GlcA in xylan^22^; as such, we speculate that a different set of enzymes may be responsible for the AGP-specific GlcAT activity. (2) The investigated genes share similar domain characteristics with functionally characterized GLCATs (ATGLCAT14A, ATGLCAT14B and ATGLCAT14C) whose function primarily involves the transfer of β-GlcA to AGPs^11^. (3) The *glcat14aglcat14bglcat14c* genetic knock-out mutants resulted in only 40% reduction in GlcA content in AGP isolates^23^. Unlike the complete loss of GlcA in gux1/2/3 triple mutant, the 40% loss of GlcA in *glcat14aglcat14bglcat14c* triple mutant may suggest the possible involvement of other putative *GLCAT* genes in the plant GT14 family in AGP GLCAT activity. In this study, we focused on a family of enzymes called GLCATs that are involved in the transfer of GlcA to AGPs for two reasons. First, GlcA is the only acidic sugar decorating the large arabinogalactan (AG) sugar chains and is known to bind to calcium in a pH-dependent manner with functional ramifications^18^. Given the important role of calcium in plant growth and developmental processes, the understanding of the evolution of this gene family will provide key insights that could be exploited for use in increasing plant biomass in bioenergy crops. Second, previous work showed that the addition of extra amounts of GlcA to commercially available gum arabic improved its emulsification properties^24^. This offers the opportunity of developing new gum arabic variants useful in the design of novel AGP-based products. To this end, we identified and characterized putative *GLCAT* gene family members in 14 plant genomes to gain insight into their evolution, sequence diversification, functional similarities/differences, and patterns of gene expression.

Gene families include genes that share similar cellular functions and commonly arise as a result of gene or genome duplication events. Gene duplication and deletion are generally considered important evolutionary mechanisms that give rise to phenotypic diversity^6^. The number of putative *GLCAT* gene family members in the investigated land plant species are comparable to an earlier report^21^ and ranged from two *GLCAT* genes in *Selaginella moellendorffii* to 22 *GLCAT* genes in soybean (Supplementary Table S1). This may be an indication of the extent of gene family expansion of *GLCATs* across evolutionary time, conceivably driven by forces of natural selection, coupled with the demand to adapt to a life on land.

Conserved regions among gene family members across species can identify functionally important motifs. Many GTs require divalent metal ions, commonly Mn^2+^ or Mg^2+^, for catalytic activity^17^ which bind to DXD motifs. In Arabidopsis, ATGLCAT14A showed full activity in the absence of Mn^2+^ or Mg^2+^; however, the addition of EDTA (> 5 mM) inhibited its activity. Apparently, this suggests that other divalent metal ions may be involved in enzyme activity^11^. In this study, we identified a moderately conserved motif, X/DWD/X among putative *GLCAT* gene family members with 71 out of 161 sequences having the DWD motif, including a bryophyte and a lycophyte. Representative sequences of the mammalian *O-*β-xylosyltransferases (XT-I, XT-II), belonging to the GT14 family, possesses a highly conserved and functionally characterized DXD motif^20^ that is absent in plant GLCATs and an uncharacterized X/DWD motif common to plants and mammals (Supplementary Fig. S1). Gotting et al.^20^ argued that the DWD motif is unique in the sense that no GT families investigated so far have an aromatic amino acid, tryptophan (W), at the central position of the consensus motif, and this observation has been reported earlier^25^. Interestingly, an investigation into the role of the tryptophan residue demonstrated that alterations of the tryptophan (W) residue in the DWD motif in the human XT-1 protein to either a neutral, basic, or acidic amino acid resulted in a > 60% reduction in enzyme activity^20^. Although, this functionally important DWD motif in human XT-1 does not align with the Arabidopsis GLCAT sequences in this study (Supplementary Fig. S1), we hypothesize that the highly conserved W residue in the X/DWD motif shared by all species may have been evolutionarily selected to play either a functional or structural role that promotes GLCAT catalytic activity in plants especially when this is the only X/DWD/X motif present among plant GT14 family members. Site-directed mutagenesis studies aimed at discovering the role of this conserved W and the flanking aspartate residues in the DWD motif could address this hypothesis.

Phylogenetic analysis provides a method for assessing homology and inferring relationships among genes. Ten clades (A-J) were identified in a phylogenetic analysis of all putative *GLCAT* gene family members investigated across plant species (Fig. 2a). Surprisingly, we discovered that clade E family members share a common ancestor before the divergence between higher and lower land plants. It is conceivable that based on the phylogenetic analysis, *Pp3c10_2430v3.10* and *Pp3c14_2510v3* found in clade E played key roles in expansion of the *GLCAT* genes into dicots and monocot grasses (Fig. 2a). Dicot specific phylogenetic analysis showed two distinct phylogenetic clades (1A and B; 2A–F), each having species representatives in each subclade (Fig. 2b). With the exception of the putative *GLCAT* genes in *Amborella trichopoda* (Supplementary Fig. S6), no single clade was found to consist of *GLCAT* genes from a single species of monocot grasses and dicots, suggesting that the putative GLCATs in *Amborella trichopoda* evolved prior to the emergence of putative GLCATs in angiosperms (Dicot and grasses).

*GLCAT* gene structure was highly conserved with most species having 4 exons and 3 introns in 97% of the sequences which includes lower and higher land plants. Similarly, the average number of amino acids in each species ranged from 360–450 amino acids (Supplementary Table 1). The above observations showed that the genetic architecture of *GLCAT* gene family members in lower and higher land plants appears to be evolutionarily conserved. In addition, we observed that the same gene architecture (4 exons, 3 introns) is present in *Amborella trichopoda* and the lower land plants examined (a bryophyte and a lycophyte). It is therefore conceivable that this gene structure originated from the shared common ancestor between the lower and higher land plants, possibly prior to the movement of plants from aquatic to terrestrial life.

Plant genomes contains a variety of structured patterns that are conserved and can be used to discover putative functions of gene family members. We identified 9 sequence blocks (blocks 1–7, 11 and 12, hence referred to as GLCAT motifs) localized in the GLCAT domain. Eight sequence blocks (blocks 1–6, 8 and 9) are present in 95% of the GLCAT sequences; however, blocks 8 and 9 are in the N terminal and C terminal regions flanking the GLCAT domains respectively (Supplementary Fig. S5). Surprisingly, block 2 which contains the highly conserved W residue of the DWD consensus motif is present in all the sequences except in *Arabidopsis lyrata* (*AL4G34150*), which after further investigation demonstrated weak sequence similarity with its closest homolog *AtGLCAT14C* (Fig. 4a). Despite the fact that other *Arabidopsis thaliana GLCAT* gene family members showed high sequence similarity with *Arabidopsis lyrata* gene family members as revealed by phylogenetic analyses (Fig. 2b), *AL4G34150* and *AtGLCAT14C* share two things in common: (1) they both have their exon–intron structure conserved and reside in the same phylogenetic clade (clade E) with *P. patens* (Fig. 2a) and (2) they both lack some blocks found in the GLCAT domain (*AtGLCAT14C* lacks blocks 11 and 12, while *AL4G34150* lacks blocks 2, 4, 5, 7 and 11). Although, *AtGLCAT14C* (*At2g37585*) has in-vitro GLCAT activity^24^, it is unknown whether its closest homolog in *Arabidopsis lyrata* has such GLCAT activity. This raises some interesting questions. For example, since ATGLCAT14C lacks blocks 11 and 12 in the GLCAT domain, could it be that sequence blocks 11 and 12 are not critical to GLCAT activity in vivo? Does its closest homolog *AL4G34150*, which lacks several blocks in the GLCAT domain and lacks the highly conserved W residue in the DWD consensus motif possess GLCAT activity? We speculate that generating and expressing constructs that lack one or more of these motifs in the GLCAT domain and testing them in an in-vitro assay will provide answers to these questions.

Previous research reported that positively selected genes are more likely to interact with each other than genes not under positive selection^26^. Using the site model of BI analysis, ω values identified amino acid residues under positive selection (*p* < 0.05). The branch model did not identify a branch under positive selection, while the branch-site model identified some sites but with significance values greater than 0.05 (Supplementary Table S3). The inability to detect positively selected sites (*p* > 0.05) in the branch-site model is indicative of functional similarity among AtGLCAT14A orthologs in both lower and higher land plants. Similarity in the genetic architecture, the conserved GLCAT domain and the shared phylogenetic clades of *GLCAT* genes may be an evolutionary pattern that is necessary for GLCAT activity in-vivo. It is well documented that the *cis*‐regulatory elements are seen as the most likely target for the evolution of gene regulation since the modular nature of *cis*‐regulatory elements largely frees protein coding regions from deleterious pleiotropic effects^27^. We are, however, not certain that this is the case in GLCATs since we did not investigate the role of *cis-*regulatory sites and their contribution to gene function in the investigated genomes.

The availability of high throughput techniques has led to the release of large amounts of data that could be mined to understand gene expression of candidate genes of major crops. Variations in cell wall composition are linked to differential gene expression patterns in different plant tissues^28,29^. In the species investigated, varying degrees of gene expression of *GLCAT* gene family members were observed across developmental stages and anatomical parts. Similarly, gene expression profiles in the anatomical parts of Arabidopsis, rice and soybean showed that most of the putative *GLCAT* gene family members have moderate to high expression values during the seedling stage, primarily in the elongation and maturation zones of the radicle (Fig. 5a,c,e). This might be reflective of the extensive cell wall modifications necessary for radicle growth. We investigated the gene expression pattern of all *GLCAT* gene family members and found *AtGLCAT14A* (AT5G39990) homologs in rice (*LOC_Os12g44240.1*) and soybean (*Glyma. 13G065900*) to be consistently expressed across all anatomical parts and may reflect conserved gene regulatory processes. In contrast, varying degrees of gene expression were observed across developmental stages and may suggest some variation of GlcA abundance in their polysaccharide chains at specific stages of development^24^.

Radicle emergence from the seed is a highly regulated process that involves discrete and coordinated changes in plant cell wall extensibility and rearrangements of its components^30^. The available data in GENEVESTIGATOR allows us to investigate the gene expression pattern of Arabidopsis and soybean during germination. While there are differential expression patterns across putative *GLCAT* gene family members in both species, there were some parallels in their temporal expression patterns. Notably, some genes in Arabidopsis and soybean were significantly up-regulated (fold change > 2) across all time points (Fig. 6a,b). Some *GLCATs* genes have low or no expression across all or some of the time points in Arabidopsis and soybean. These two observations underscore the importance of certain *GLCAT* gene family members in germination as growing cells need to produce tailored polysaccharide-rich cell walls essential to determining overall plant growth and biomass.

As sessile organisms, plants must cope with the onslaught of abiotic stresses, such as cold, hypoxia, and extreme temperatures. As most of the *GLCAT* genes were significantly up-regulated during germination, some of the *GLCAT* gene family members were significantly down-regulated in response to abiotic stress. We observed that the expression of most, if not all of the *GLCAT* genes in Arabidopsis were either unchanged or severely down-regulated (fold change < 2) in response to anoxia, hypoxia and heat stress (Fig. 7a). This might be as a result of the need to divert resources from growth to the expression of genes involved in adaptation and survival. In all the rice genotypes examined, we observed a differential response to cold stress; while most *GLCAT* genes were down-regulated, *LOC_Os03g16890* gene was consistently up-regulated in response to cold (4 °C) but down-regulated as the temperature increased to 29 °C in the LTH and IR29 rice genotypes. Notably, *LOC_Os12g44240* (an *AtGLCAT14A* homolog) was consistently down-regulated in response to cold, but up-regulated as the temperature increased to 29 °C in the LTH and IR29 rice genotypes (Fig. 7b). Taken together, there appear to be differences in *GLCAT* gene expression in response to abiotic stress, with concomitant effects on plant growth and biomass production.

## Materials and methods

### Sequence retrieval and identification of *GLCAT* gene family in plant lineages

Database searching was conducted with the Phytozome database version v12.1.6 (https://phytozome.jgi.doe.gov/pz/portal.html) for 14 plant genomes including *Amborella trichopoda* using the *Arabidopsis thaliana GLCAT* gene family as queries in a BLAST search. The database search was conducted for the following investigated genomes belonging to the following species: *Selaginella moellendorffii*, *Physcomitrella patens*, *Arabidopsis thaliana*, *Arabidopsis lyrata, Brachipodium distachyon, Citrus sinensis, Glycine max, Gossypium raimondii, Oryza sativa*, *Populus trichocarpa*, *Solanum lycopersicum*, *Sorghum bicolor*, *Vitis vinifera,* and *Zea mays.* For the identification and analysis of *GLCAT* genes in the plant genomes, genomic sequences, coding sequences (cds) and peptide sequences were downloaded from the Phytozome version v12.1.6 database^31^ after a BLASTp 2.2.28 + search. BLASTp was performed by using *Arabidopsis GLCAT* gene members as queries to retrieve *GLCAT* gene family members of the remaining 13 investigated genomes with an *E*-value of 10^−5^. Unique *GLCAT* genes were filtered by excluding partial and redundant sequences. All identified putative GLCAT proteins were further confirmed for the presence of family specific conserved domains using NCBI’s Conserved Domain Database (CDD); (https://www.ncbi.nlm.nih.gov/Structure/cdd/wrpsb.cgi), Branch domain (PF02485) and EMBL InterProScan (https://www.ebi.ac.uk/interpro/). After confirming the presence of the conserved Branch domains (PF02485) for all putative GLCAT proteins, we further obtained gene IDs, functional annotations, chromosome locations, chromosome numbers, genomic coordinates and peptide sizes from the Phytozome database. Protein molecular weights and pI-values for the identified proteins were calculated using the ExPASy online tool^32^.

### Phylogeny, gene structure analysis, physical mapping and synteny analysis

The GLCAT protein sequences of the investigated genomes were aligned in ClustalW and illustrated using Jalview^33^. PhyML and Bayesian inference (BI) were used to construct respective phylogenetic trees. PhyML was constructed using maximum likelihood with a bootstrap value of 1000 iterations, and all positions containing gaps and missing data were excluded in order to achieve phylogenetic trees. For the BI, phylogenetic reconstruction was carried out using Bayesian Markov Chain Monte Carlo (MCMC) as implemented in BEAST software v1.5.4^34^. The analysis was carried out with the following parameters: relaxed molecular clock with an uncorrected log-normal distribution model for rate of variation, the HKY substitution model, four gamma categories and a Yule model of speciation. Three independent runs were carried out, each with 1 million MCMC generations and sampled every 1000th generation. Finally, the trees were visualized and managed in iTOL^35^.

A physical map of *GLCAT* gene members was constructed using the chromosome numbers and genomic coordinates in Mapchart 2.30^36^. Gene Structure Display Server^37^ was used to determine the number of introns and exons. Also, syntenic regions were identified between *A thaliana* and its orthologs in the investigated genomes using Circoletto^38^. The color represented the extent of similarity, blue for the lowest similarity, followed by green, orange and red, showing the increasing extent of similarity with increasing bit score. Specifically, blue for the first (i.e. worst) 25% of the maximum bitscore, green for the next 25%, orange for the third, and red for the top (i.e., best) bitscores of between 75 and 100% of the maximum bitscore^38^.

### Motif identification in GLCAT sequences in plant genomes

For motif identification, conserved motifs among GLCATs were identified using the MEME program^39^ with the following parameters: number of repetitions = zero or one, maximum number of motifs = 6, and optimum motif width constrained between 6 and 50 residues.

### Positively selected sites in GLCATs and their putative biological significance

For the identification of positively selected sites, we considered ATGLCAT14A (AT5G39990) orthologs since this protein is the only GLCAT that has been extensively characterized^11^ and would be a more reliable estimates of selection pressure. We performed a strict statistical analysis using the CodeML program in the EasycodeML software^40^ using branch model, site model, and branch-site model^41^ in a run based on the non-synonymous (dN) and synonymous (dS) nucleotide substitution rate ratio (dN/dS) or ω. If ω > 1, then there was a positive selection on some branches or sites; ω < 1 suggests a purifying selection (selective constraints); and ω = 1 indicates neutral evolution. The parameter estimates (ω) and likelihood scores^42^ were calculated for three pairs of models. These were M0 (one-ratio) versus M3 (discrete), M1a (nearly-neutral) versus M2a (positive-selection), and M7 (beta) versus M8 (beta&ω)^43^. The LRT^44^ was used to compare the fit to the data of two nested models, which measured the statistical significance of each pair of nested models based on the estimated *p* values^45^. A significantly higher likelihood of the alternative model compared to the null model suggests positive selection (ω > 1). Similarly, we also implemented the branch model to select the statistically significant “foreground branch” presumed to be under positive selection while all other branches in the tree were “background” branches (for example, making branch I foreground while branch II and III are background, Fig. 2d). The background branches share the same distribution of ω values among sites, whereas different values can apply to the foreground branch. Then, the branch-site model was applied, which further estimated the different dN/dS values among the significant branches detected by the branch model and among sites^46^. Finally, a Bayes empirical Bayes (BEB) approach was then used to calculate the posterior probabilities that a site comes from the site class with ω > 1, which when implemented in EasycodeML software, were used to identify sites under positive selection or purifying selection in the foreground group with significant LRTs^44^. Each branch group was labeled as a foreground group while other branch groups were considered background.

### Digital expression analysis of plant genomes

Publicly available mRNA-seq and Affymetrix microarray data were used to determine the expression of the *GLCAT* gene family members in *Oryza sativa*, *Arabidopsis thaliana* and *Glycine max.* Complete *GLCAT* gene expression data were only available for these three species. We used the GENEVESTIGATOR software (https://genevestigator.com/gv/) because the expression dynamics for *GLCAT* genes are useful for species comparisons under different conditions. Specifically, mRNA-seq data were used to evaluate the expression of *GLCAT* gene family members across developmental stages and anatomical parts, while the Affymetrix microarray data were used to investigate the expression of the *GLCAT* gene family members during germination and abiotic conditions. Both mRNA-seq and Affymetrix data were derived from GENEVESTIGATOR (https://genevestigator.com/gv/) and species expression values for the *GLCAT* gene members were displayed as heatmaps by GENEVESTIGATOR.

## Supplementary information

Supplementary Information.

## Data Availability

All data generated or analyzed during this study are included in this published article (and its Supplementary Information files).
