# Supplementary material for: Systems identification and characterization of β-glucuronosyltransferase genes involved in arabinogalactan-protein biosynthesis in plant genomes
Source: Sci Rep. 2020 Nov 25;10:20562. doi: 10.1038/s41598-020-72658-4 (PMC7689455; doi:10.1038/s41598-020-72658-4)

**Systems Identification and Characterization of β-Glucuronosyltransferases Genes Involved in Arabinogalactan-Protein Biosynthesis in Plant Genomes**

Oyeyemi Olugbenga Ajayi^1,2^ and Allan M. Showalter^1,2^

^1.^ Department of Environmental and Plant Biology, Ohio University, USA, 45701

^2.^ Molecular and Cellular Biology Program, Ohio University, USA, 45701

***Corresponding author**

Allan M Showalter

[showalte@ohio.edu](mailto:showalte@ohio.edu)

**Supplementary figures**

**Supplementary figure S1. Protein sequence alignment of characterized GT14 sequences from Arabidopsis, humans, mouse, rat, *C. elegans*, *Gallus gallus*, *D. melanogaster***. The N terminal region (Black boxed region) and the C terminal (green boxed region) DXD motif has been functionally characterized in human XT-1; only the C terminal (green boxed region) DXD motif is utilized for its catalytic activity as resolved by site directed mutagenesis study [24]. This functionally important motif is absent in plants. Red boxed region indicated an uncharacterized DXD motif with a conserved tryptophan residue shared by all species. Asterisk indicate conserved W.


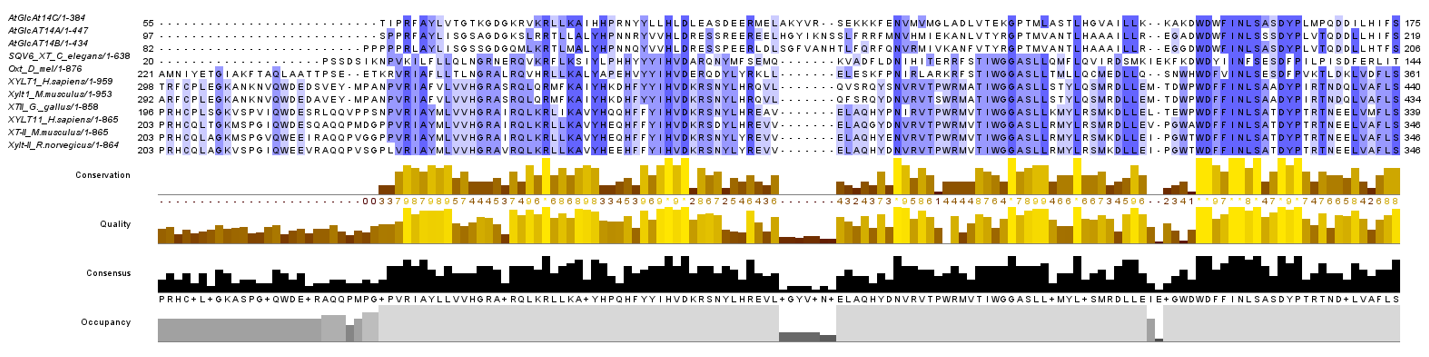

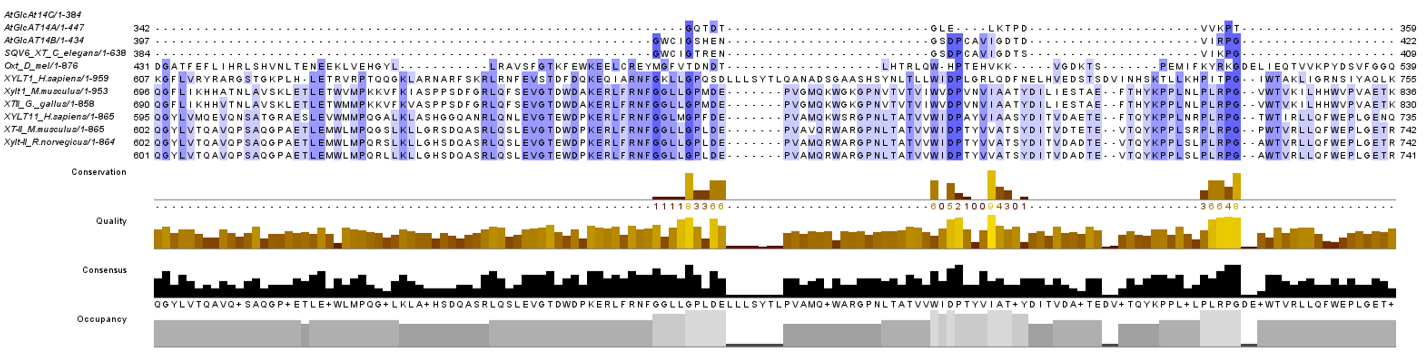


*****

*****

**Supplementary figure S2. Map showing the chromosomal locations of *GLCAT* genes in Arabidopsis thaliana, Arabidopsis lyrate, Glycine max, *Gossypium raimondii*, *Populus trichocarpa, Solanum lycopersicum*, *Sorghum bicolor*, *Brachipodium distachyon, Vitis vinifera* and *Oryza sativa*.** The number of genes on each chromosome are indicated.

*Arabidopsis lyrata*

*Arabidopsis thaliana*


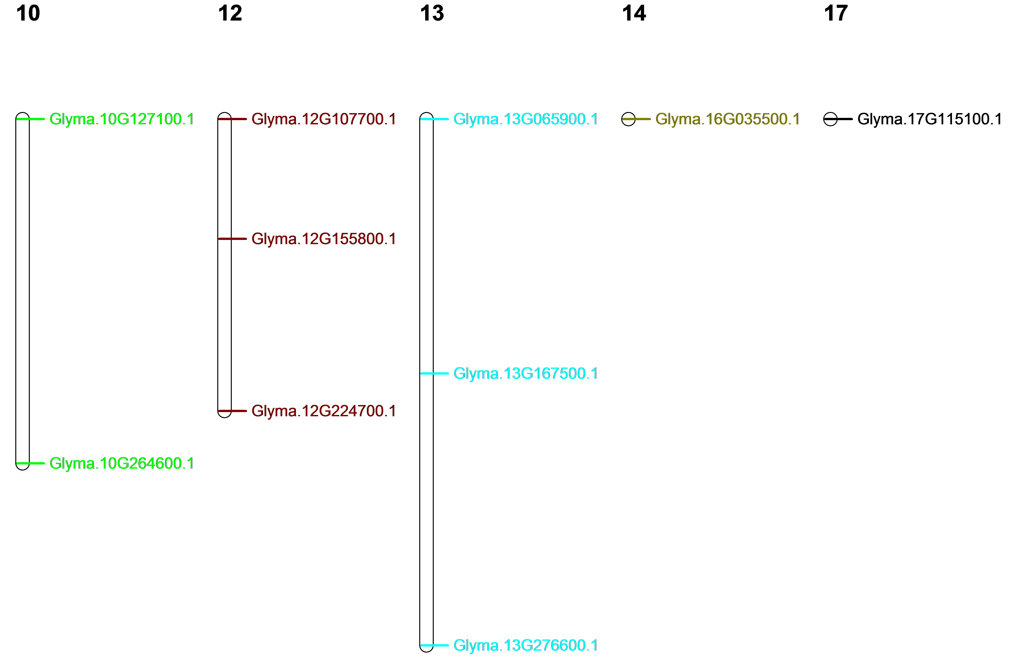

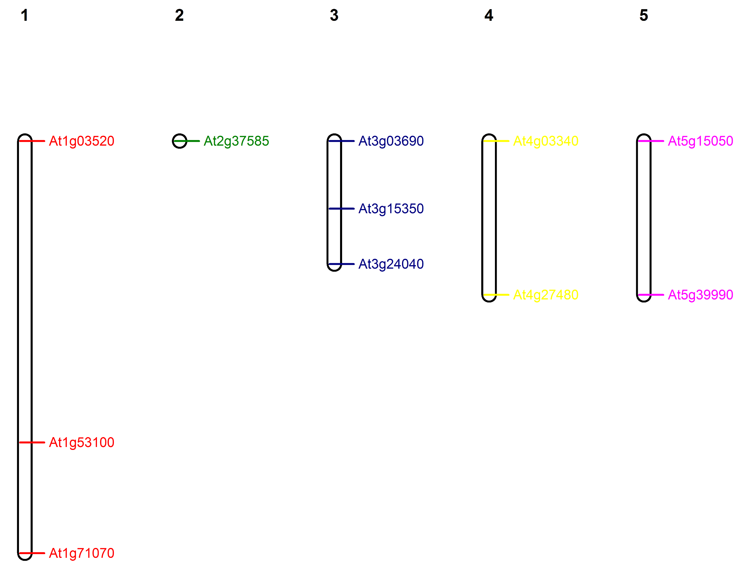

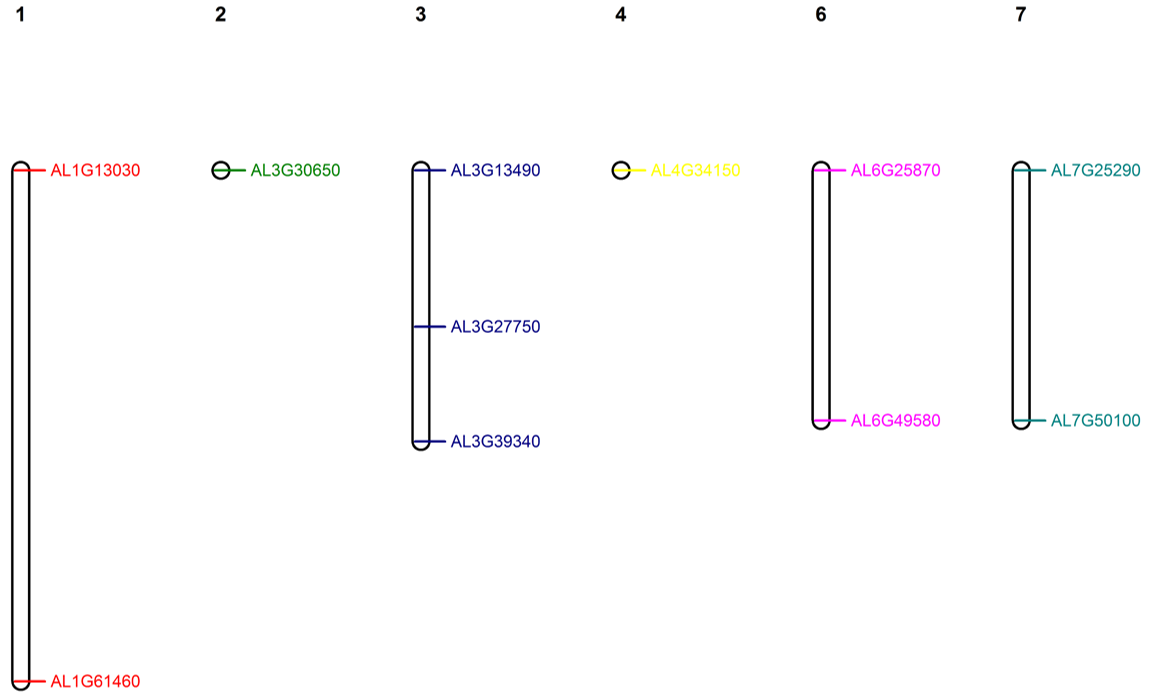

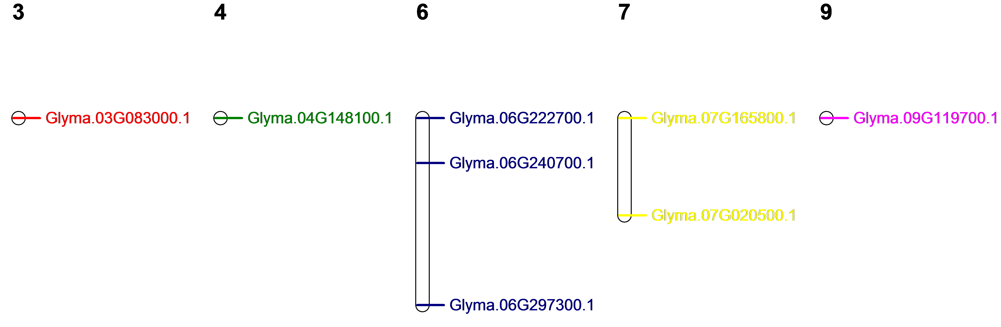


*Glycine max*


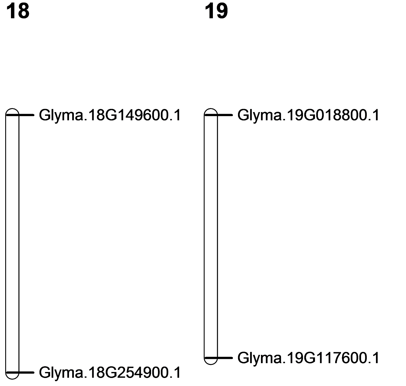

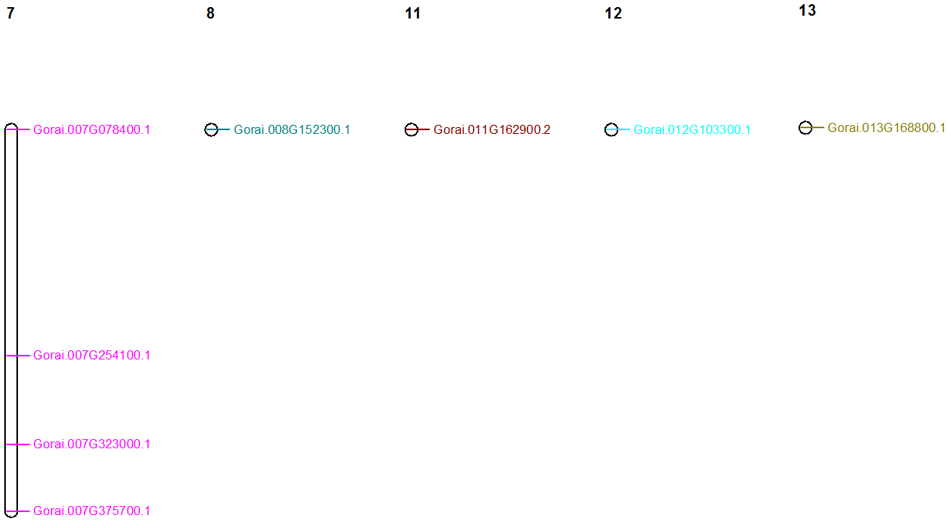

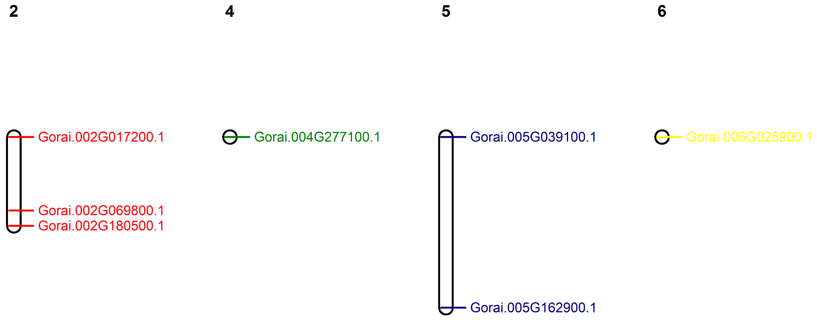

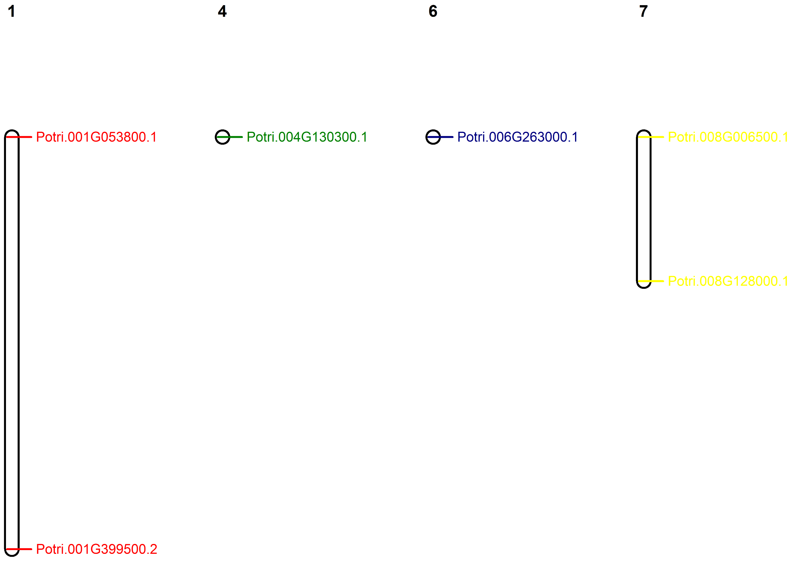

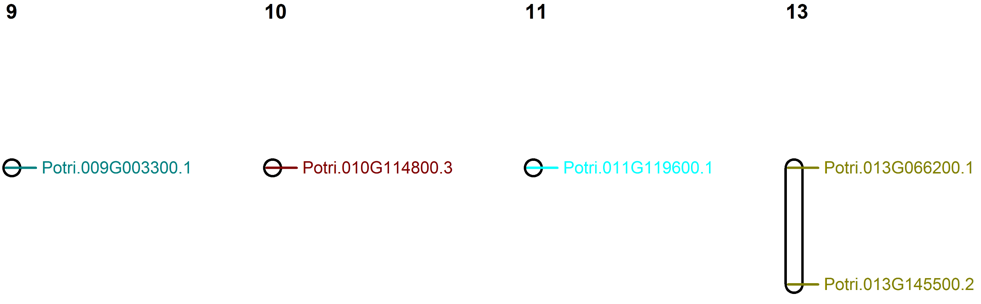

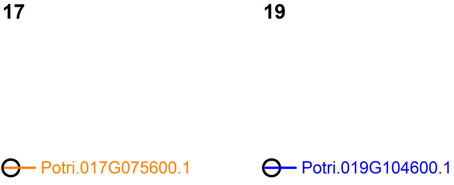


*Gossypium raimondii*

*Populus trichocarpa*


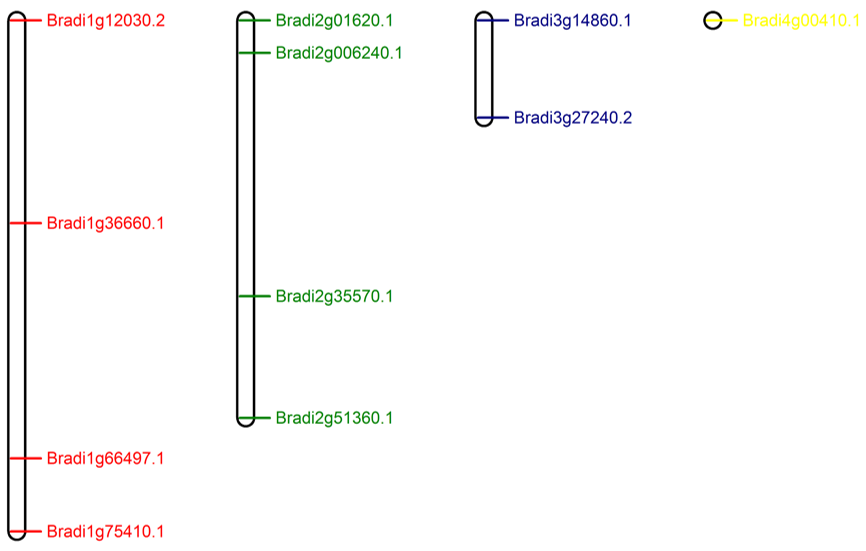

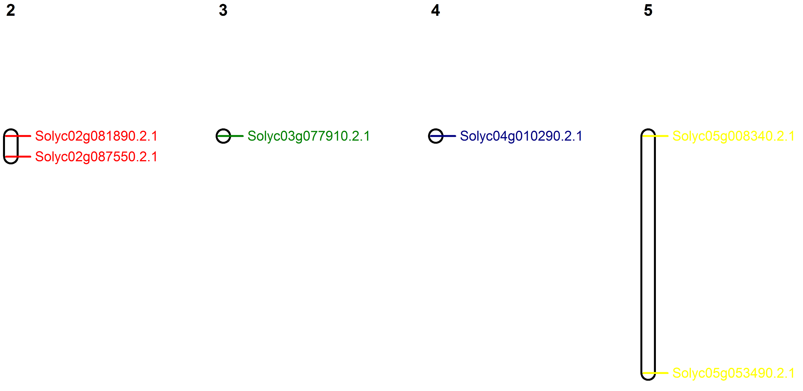

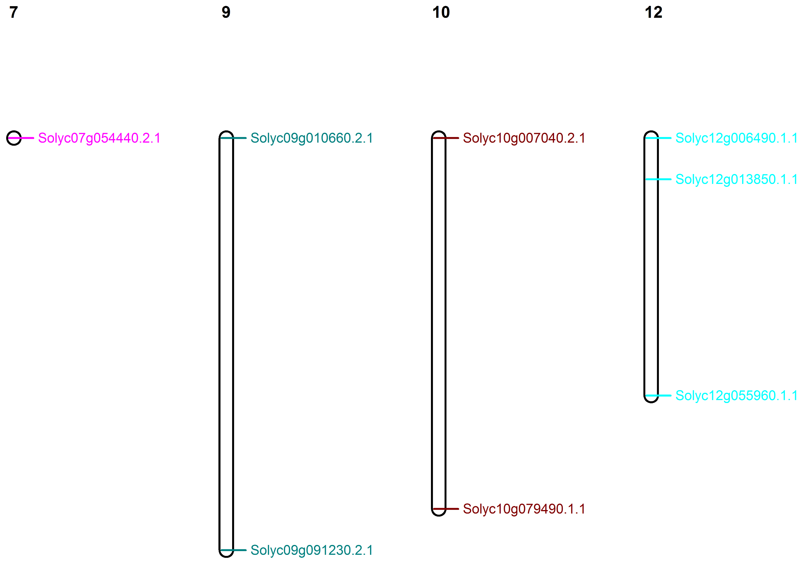

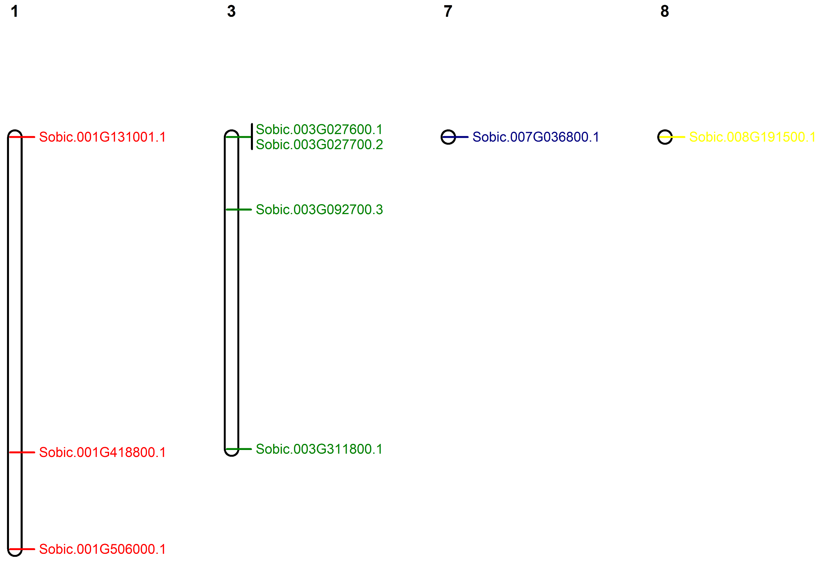

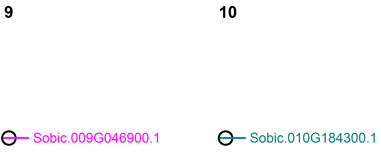


*Solanum lycopersicum lycopersicum*

*Sorghum bicolor*


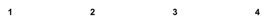


*Brachipodium distachyon distachum*


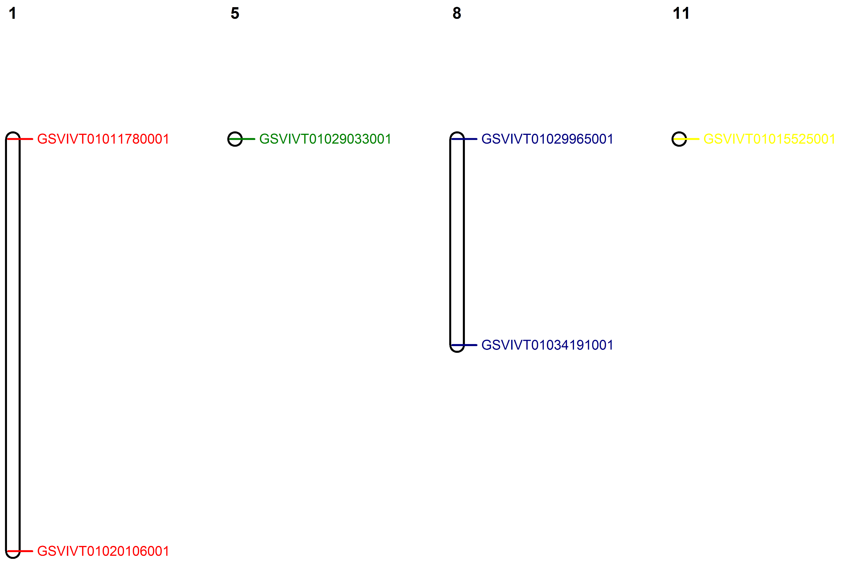

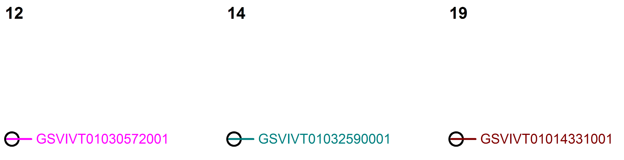

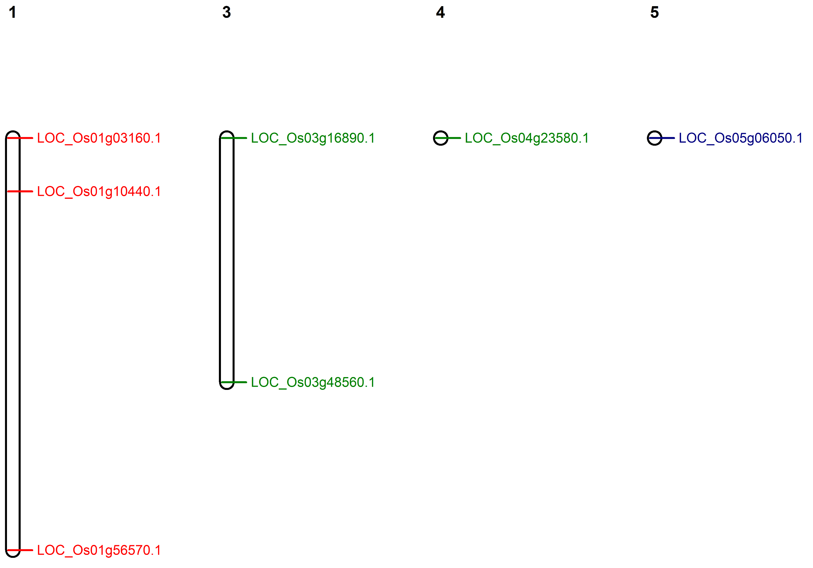

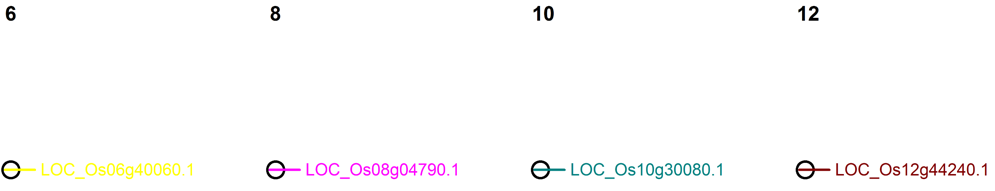


*Vitis vinifera*

*Oryza sativa*


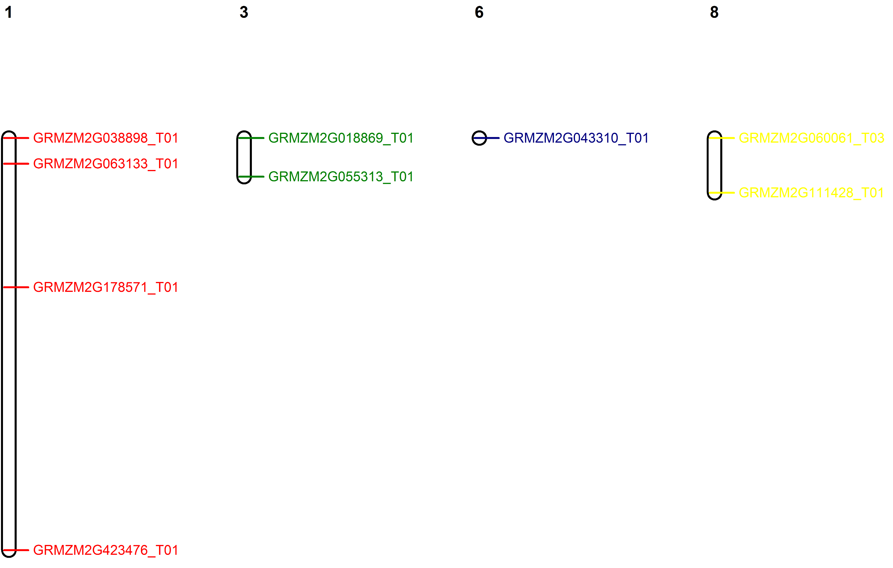


*Zea mays*


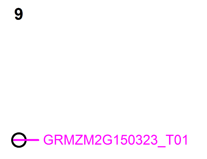


**Supplementary figure S3. Gene structure display of *GLCAT* sequences from various plant species**. Gene structure is representative of bryophytes, lycophytes and dicots (A) and monocots (B) as illustrated by using the GSDS 2.0 server [37].

*Selaginella moellendorffii*


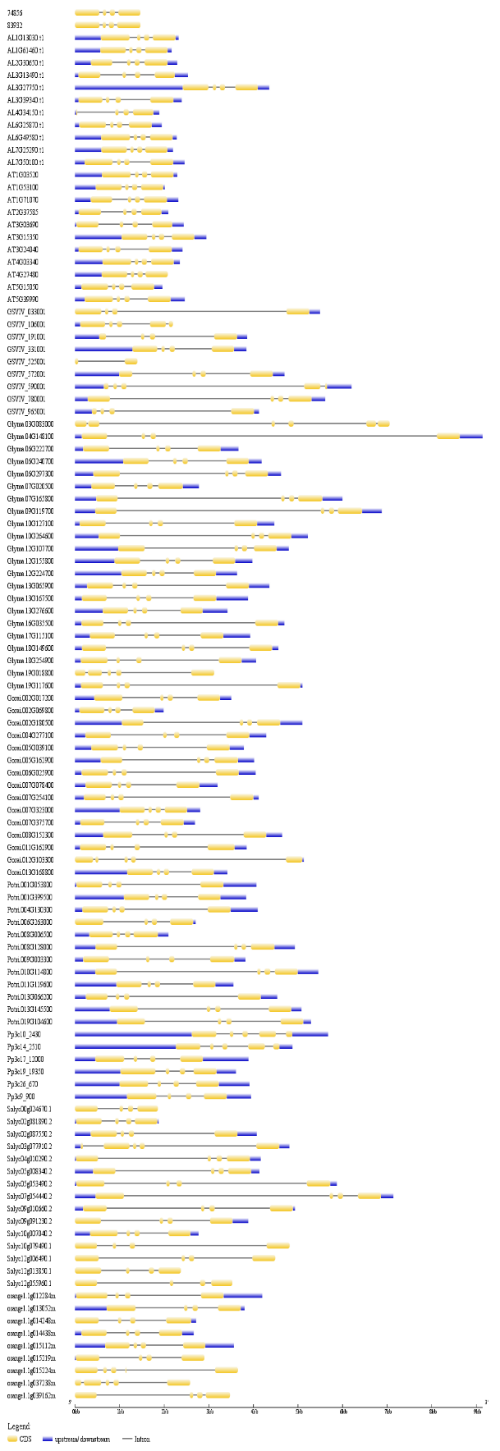


*Arabidopsis lyrata*

*Arabidopsis thaliana*

*Vitis vinifera*

*Glycine max*

*Gossypium raimondii*

*Populus trichocarpa*

*Physcomitrella patens*

*Solanum lycopersicum*

*Citrus sinensis*


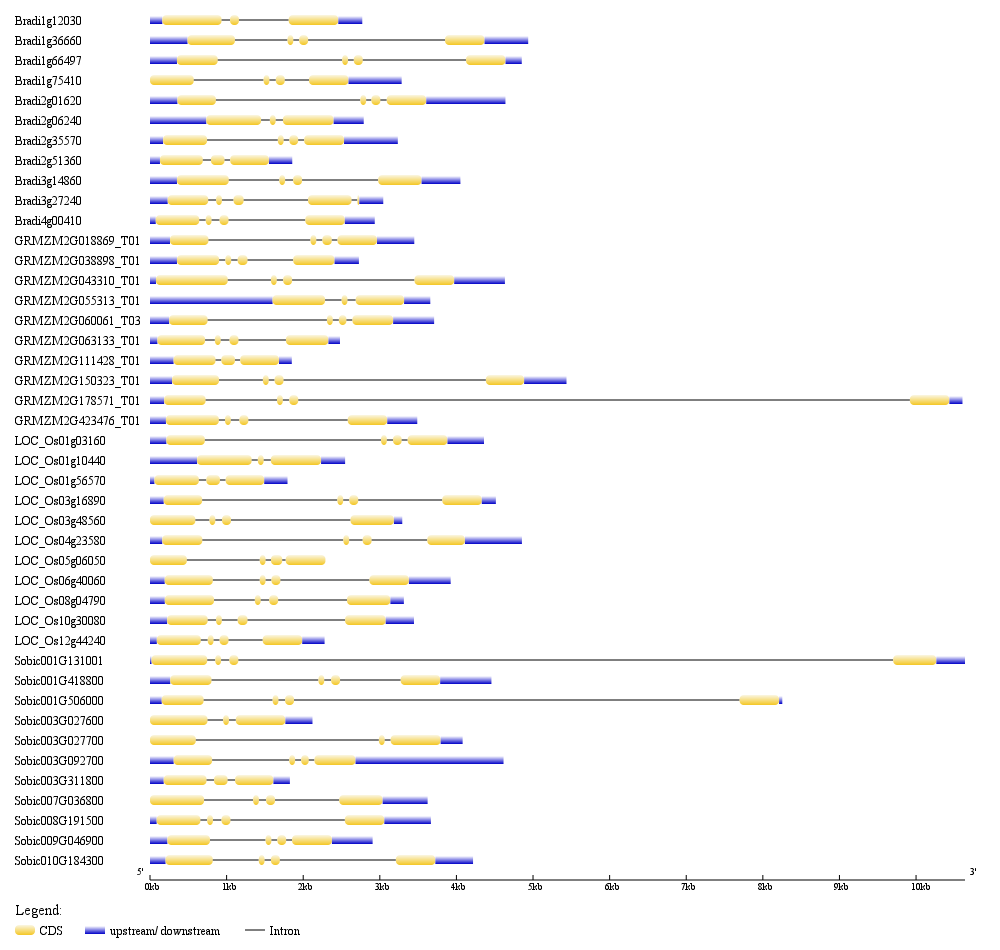


*Brachipodium distachyon*

*Zea mays*

*Oryza sativa*

*Sorghum bicolor*

**B**

**Supplementary figure S4. Synteny analysis of *GLCAT* gene families in plant species.** Synteny analysis of *Arabidopsis thaliana* with (A) *A lyrata*, (B) brachipodium, (C) citrus, (D) soybean, (E) cotton, (F) rice, (G) *P patens*, (H) Poplar, (I) Selaginella, (J) Tomato, (K) Sorghum and (L) *Vitis vinifera*.. Inside the circle, ribbons represent local alignments based on bit score, red (> 80%), orange (> 60%), green (> 40%) and blue (>20%). Ribbon width is correlated with % identity. Ribbons representing best hits are outlined and placed on top of all other ribbons. Histogram on the top of the ideograms, shows how many times each colour has hit the specific part of the sequence.


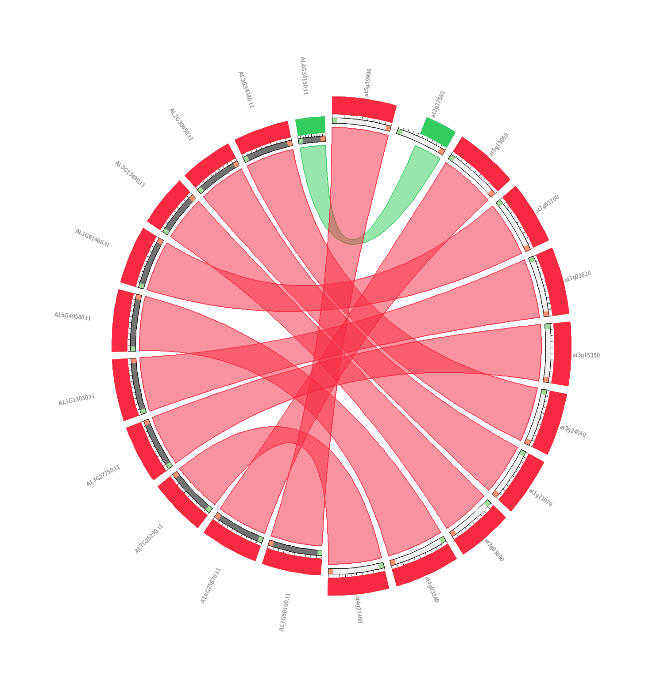

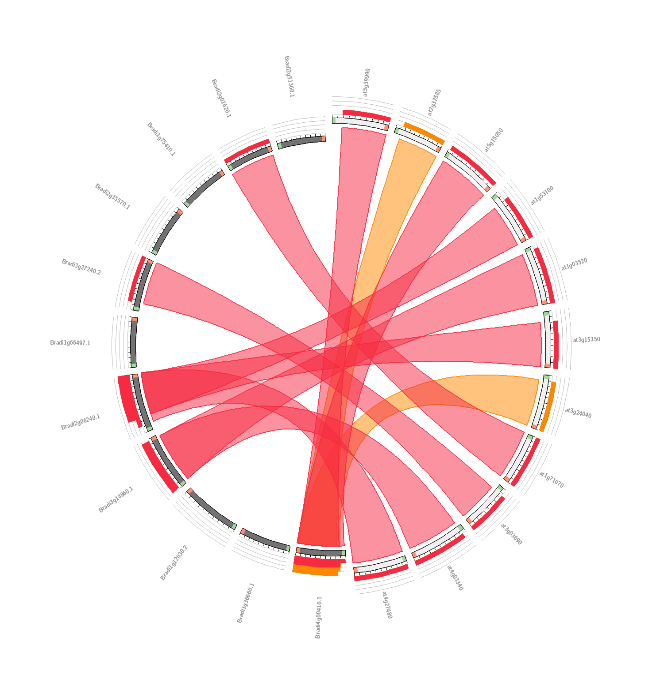


**A**

**B**


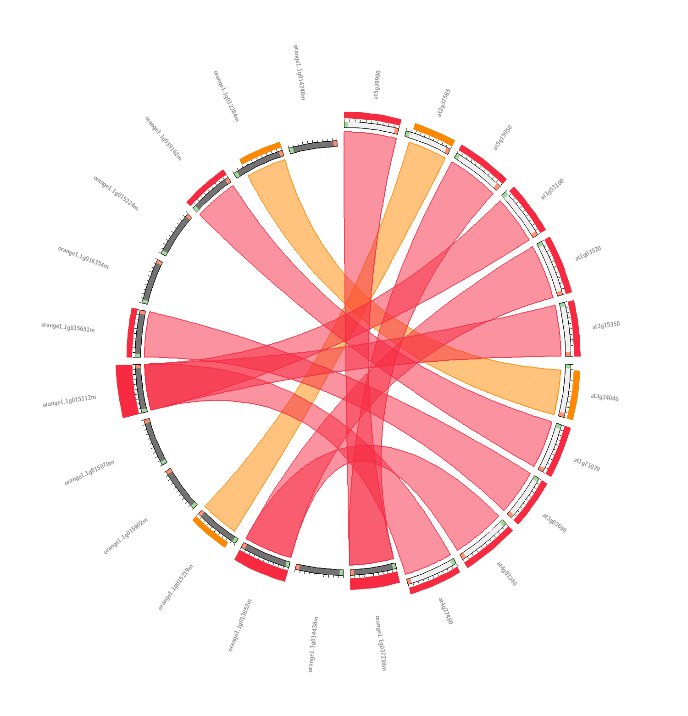


**C**


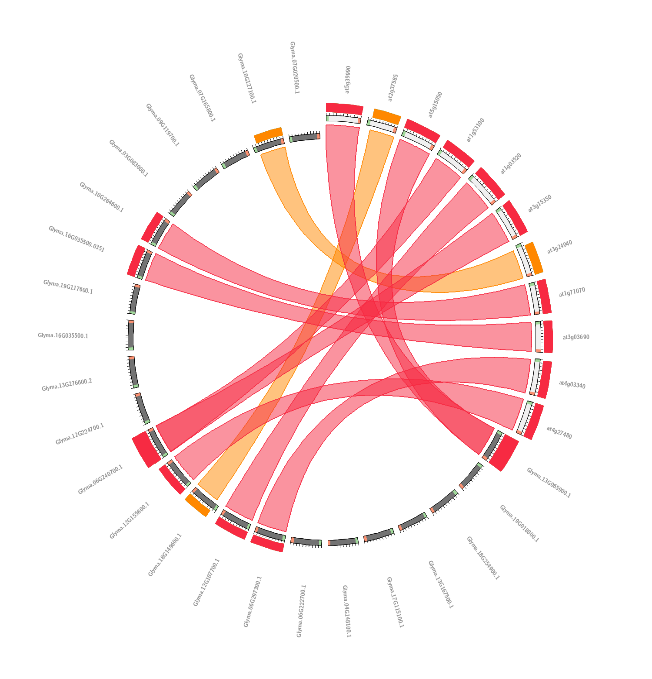


**D**


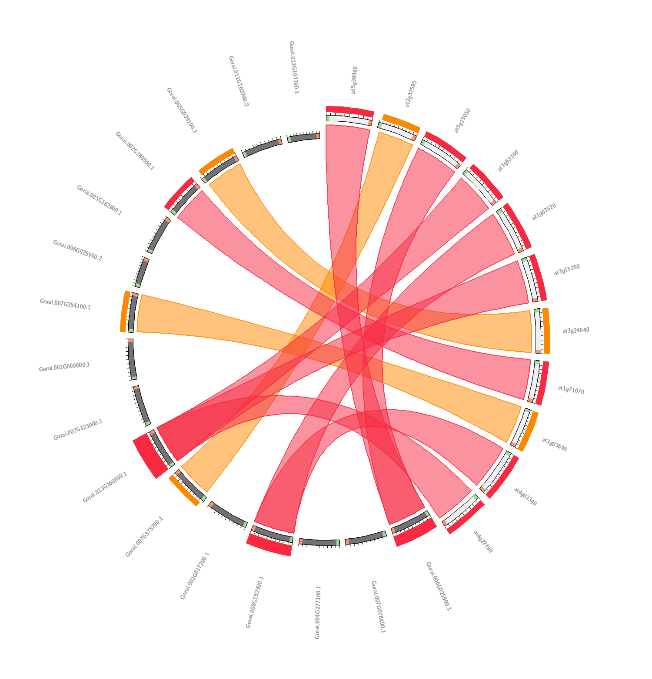

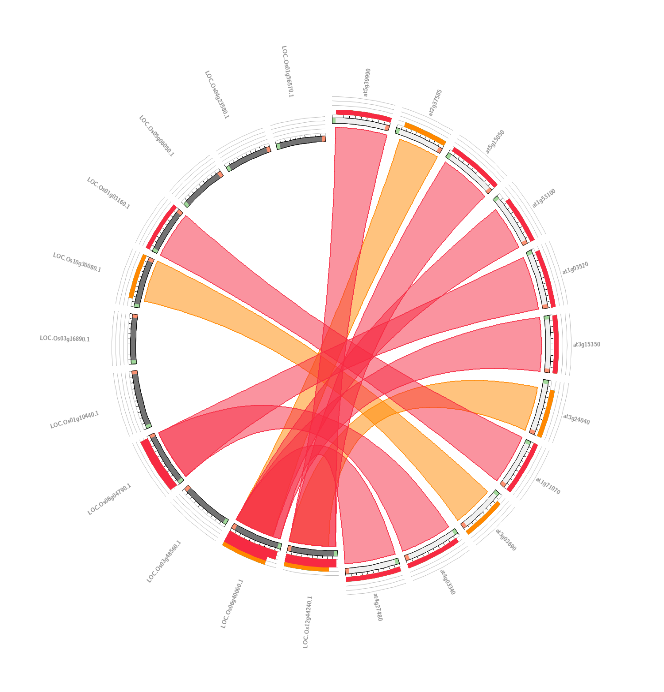


**F**


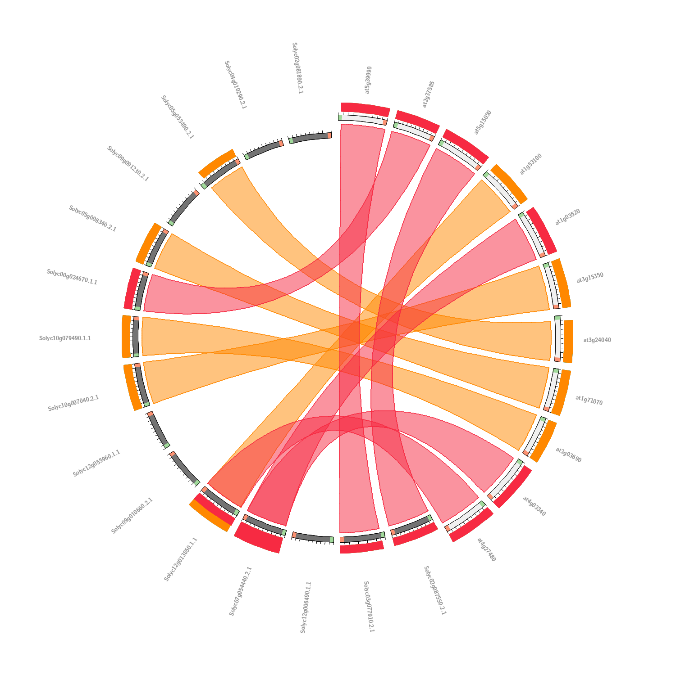

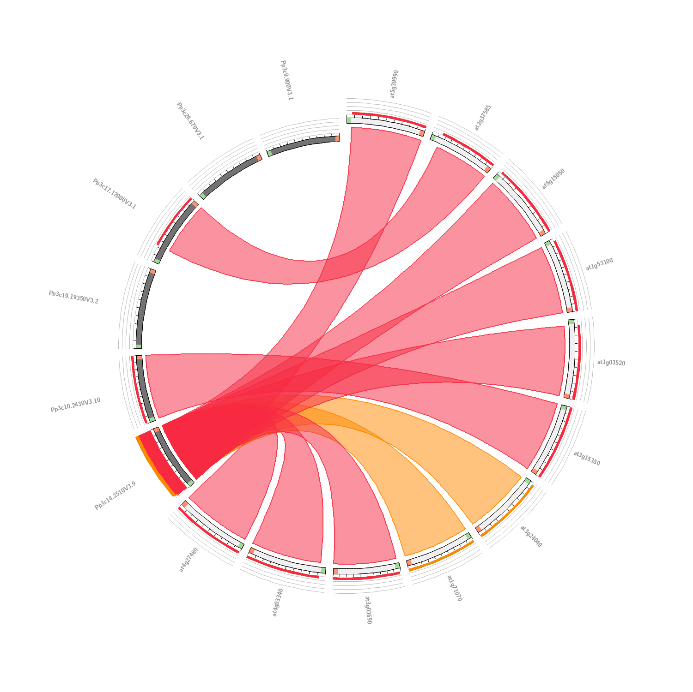


**G**


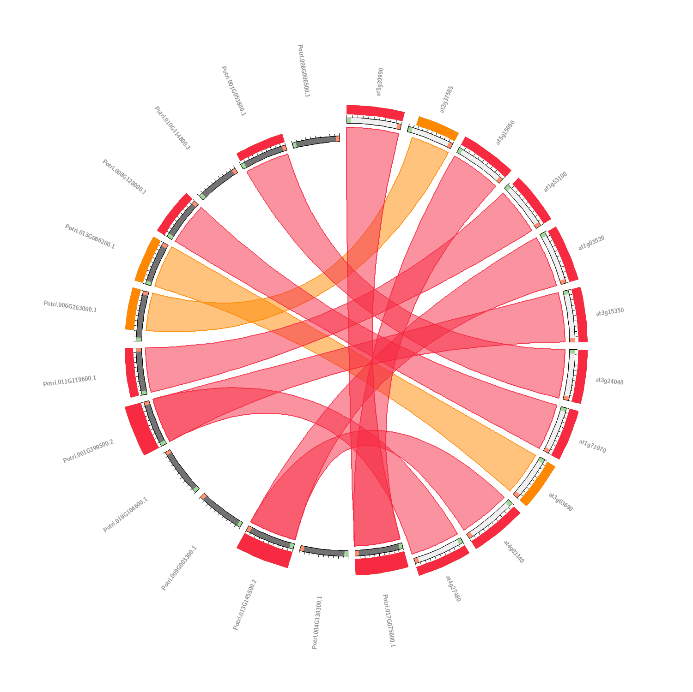


**H**


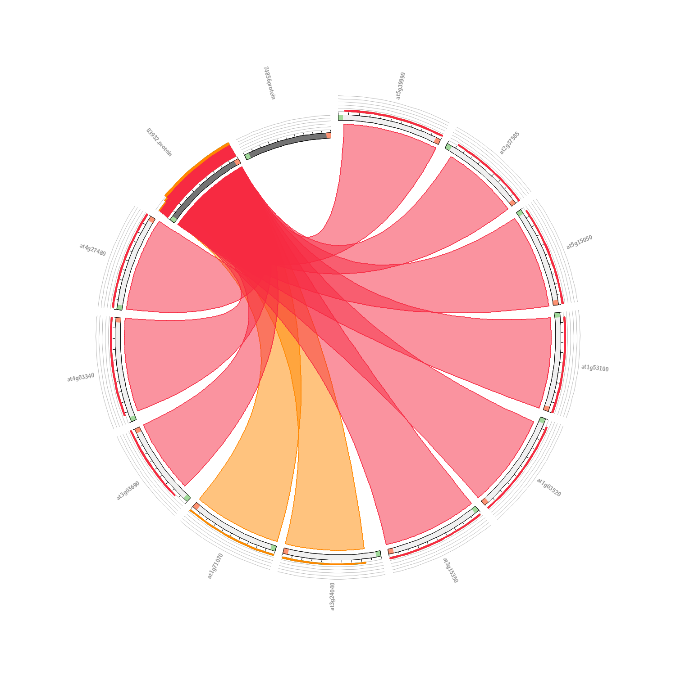


**I**

**J**


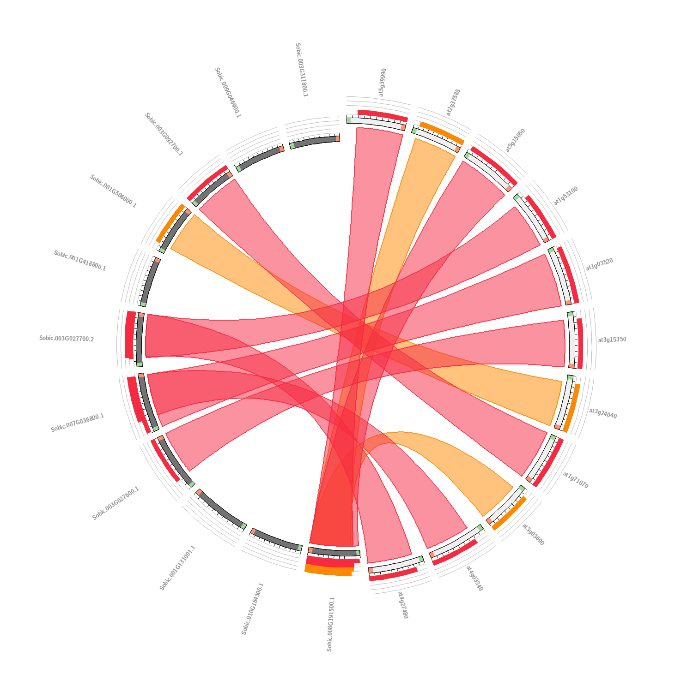


**K**


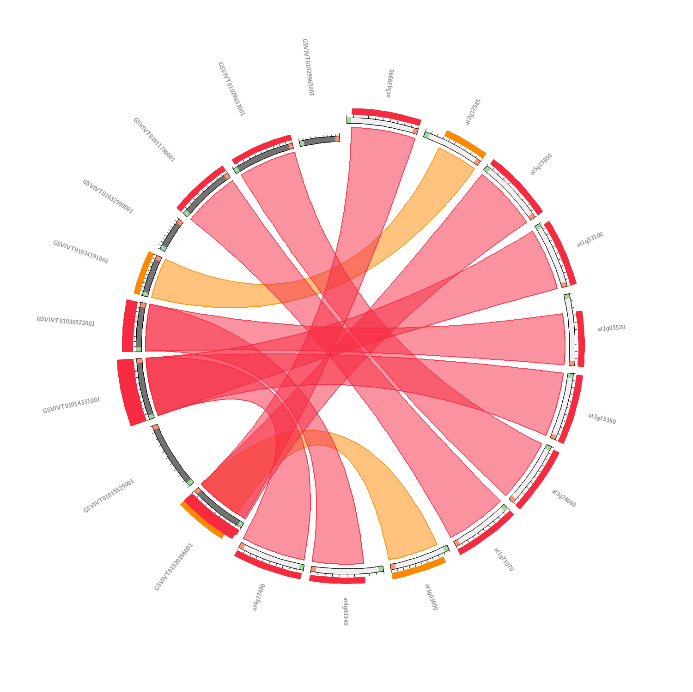


**L**

**Supplementary Fig. S5. Conserved motifs/blocks as predicted by MEME software.** These eight identified motifs are found in 95% of the GLCAT sequences derived from multiple sequence alignment of all GLCAT family members from the 14 plant genomes examined. (See Table 1 for more details.)


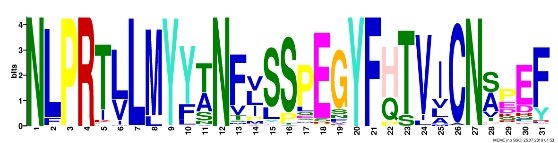

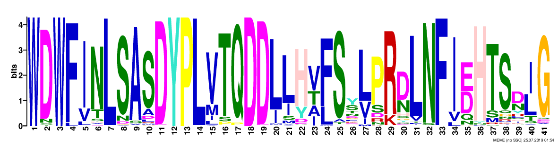

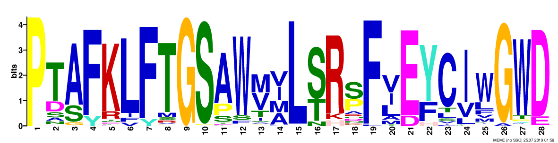

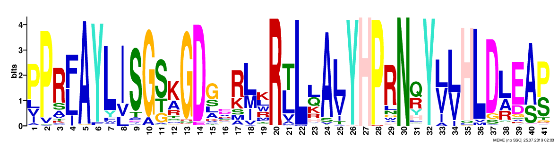

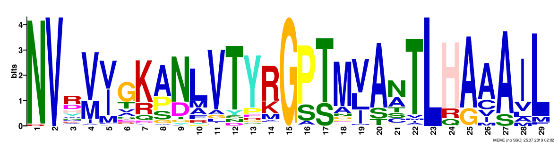

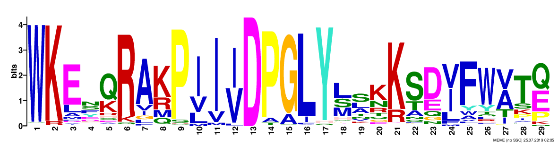


Motif 1

Motif 2

Motif 3

Motif 4

Motif 5

Motif 6


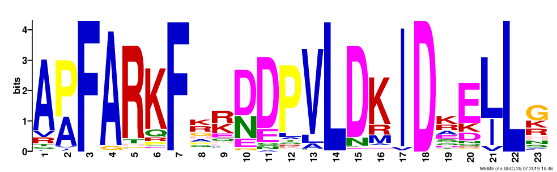


Motif 8

Motif 9


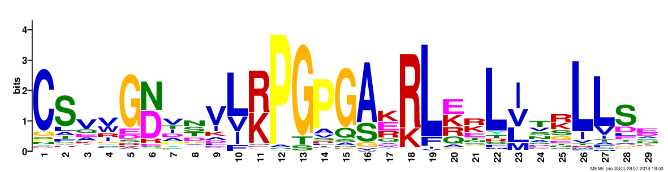


**Supplementary Fig. S6. Phylogenetic analyses of GLCATs in plant genomes using maximum likelihood method**. Figure showed the phylogenetic analyses in 14 plant species investigated in addition to putative GLCAT sequences in Amborella genome (indicated with a purple color). The eleven putative GLCAT sequences identified in the *Amborella trichopoda* genome occupy distinct nodes and notably evolved prior to the common ancestor of angiosperms. Two main evolutionary events were discovered and depicted with red and blue clades.


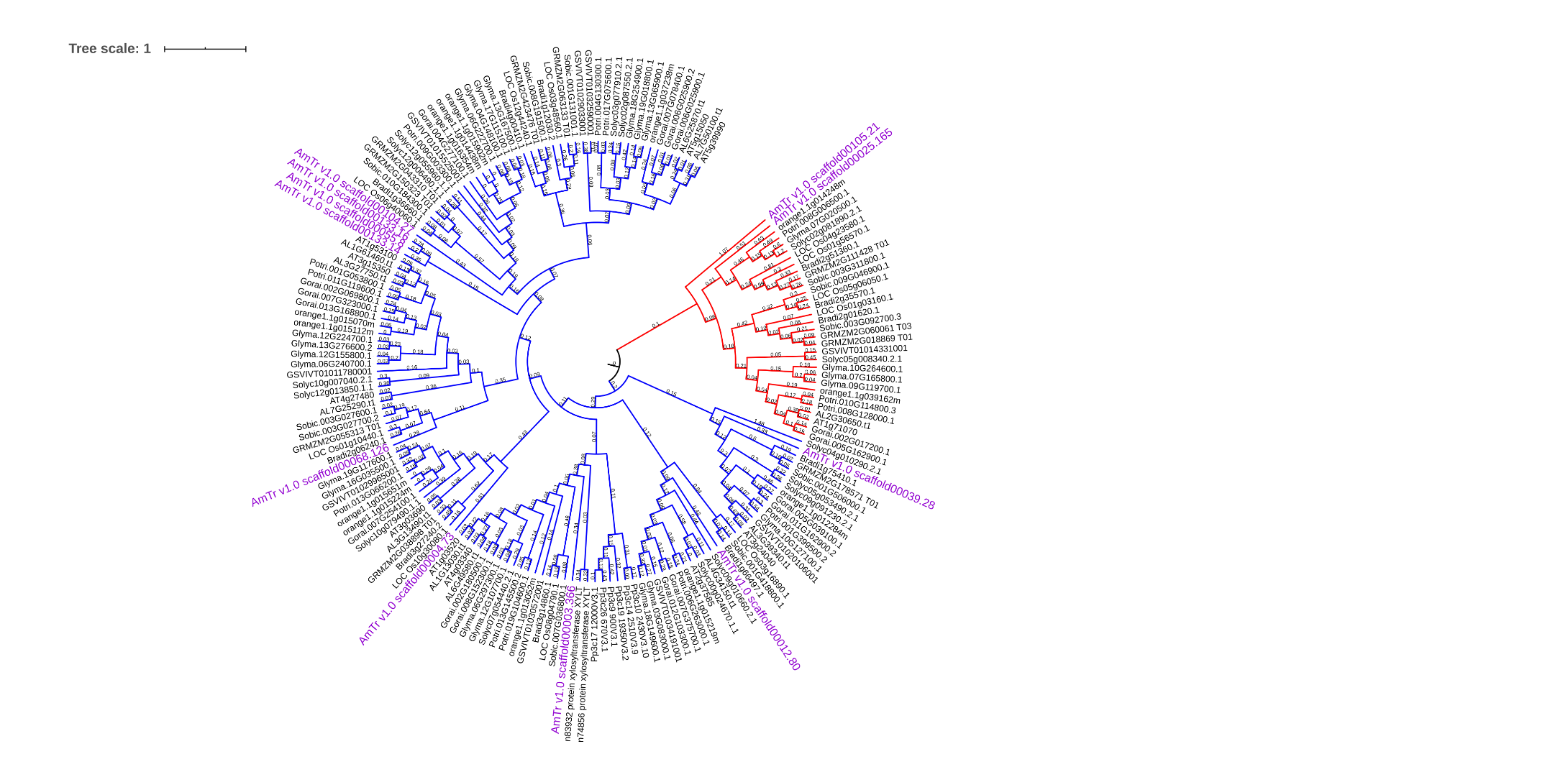

Supplement: Supplementary file 1 — Supplementary Information. [file 41598_2020_72658_MOESM1_ESM.docx]
